# Supplementary material for: Patients with nodding syndrome in Uganda improve with symptomatic treatment: a cross-sectional study
Source: BMJ Open. 2014 Nov 13;4(11):e006476. doi: 10.1136/bmjopen-2014-006476 (PMC4244396; doi:10.1136/bmjopen-2014-006476)
Supplement: Author's manuscript [file bmjopen-2014-006476.draft_revisions.pdf]

BMJ Open

Nodding syndrome patients in Uganda improve with symptomatic treatment; a cross sectional study

Journal:	*BMJ Open*
Manuscript ID:	bmjopen-2014-006476
Article Type:	Research
Date Submitted by the Author:	28-Aug-2014
Complete List of Authors:	Idro, Richard; Mulago Hospital/Makerere University, Department of Paediatrics and Child Health; Oxford University , Nuffield Department of Medicine<br>Namusoke, Hanifa; Mulago Hospital/Makerere University, Department of Paediatrics and Child Health<br>Abbo, Catherine; Mulago Hospital/Makerere University, Department of Psychiatry<br>Mutamba, Brian; Mulago Hospital/Makerere University, Department of Psychiatry; Butabika Hospital, Department of Psychiatry<br>Kakooza, Angelina; Mulago Hospital/Makerere University, Department of Paediatrics and Child Health<br>Opoka, Robert; Mulago Hospital/Makerere University, Department of Paediatrics and Child Health<br>Musubire, Abdu; Mulago Hospital/Makerere University, Department of Internal Medicine<br>Mwaka, Amos; Mulago Hospital/Makerere University, Department of Internal Medicine<br>Opar, Bernard; Ministry of Health Headquarters, Department of Clinical Services
**Primary Subject Heading**:	Neurology
Secondary Subject Heading:	Global health
Keywords:	Paediatric neurology < PAEDIATRICS, Epilepsy < NEUROLOGY, Developmental neurology & neurodisability < PAEDIATRICS

SCHOLARONE™
Manuscripts

Treatment Outcomes of Nodding Syndrome in Uganda

Nodding syndrome patients in Uganda improve with symptomatic treatment; a cross sectional study

Richard Idro[1, 2], Hanifa Namusoke H[1], Catherine Abbo C[3,4], Brian B Mutamba[3,5], Angelina Kakooza-Mwesige[1,6], Robert O Opoka RO[1], Abdu K Musubire[7], Amos D Mwaka AD[7], Bernard T Opar[8]

[1]Department of Paediatrics and Child Health, Mulago hospital/Makerere University College of Health Sciences, Kampala, Uganda

[2]Centre for Tropical Medicine, Nuffield Department of Medicine, University of Oxford, UK

[3]Department of Psychiatry, Mulago hospital/Makerere University College of Health Sciences, Kampala, Uganda

[4]Red Cross War Memorial Children's Hospital, Division of Child and Adolescent Psychiatry, University of Cape Town, South Africa.

[5]Butabika National Referral hospital, Kampala, Uganda

[6]Astrid Lindgren Children's Hospital, Neuropediatric Research Unit, Karolinska Institutet, Sweden

[7]Department of Internal Medicine, Mulago hospital/Makerere University College of Health Sciences, Kampala, Uganda

[8]Ministry of Health Headquarters, Kampala, Uganda

Key words

Nodding syndrome, epilepsy, treatment, outcome

Word count

Abstract 299

Full text 3,449

Running title

Treatment Outcomes of Nodding Syndrome in Uganda

Correspondence

Dr Richard Idro

Department of Paediatrics and Child Health, Mulago hospital/Makerere University College of Health Sciences, P.O Box 7072, Kampala, Uganda

Tel: +256 414 531875

Email: ridro1@gmail.com

For peer review only - http://bmjopen.bmj.com/site/about/guidelines.xhtml

For peer review only

Treatment Outcomes of Nodding Syndrome in Uganda

ABSTRACT

Objectives

Nodding syndrome (NS) is a poorly understood neurologic disorder affecting thousands of children in Africa. We introduced a symptomatic treatment intervention in Uganda in March 2012. This included sodium valproate for seizures, management of behaviour and emotional difficulties, nutritional and physical rehabilitation. We assessed the clinical and functional outcomes of the intervention after at least 12 months of implementation.

Design

This was a cross-sectional study of a cohort of patients with NS receiving the specified intervention. We abstracted pre-intervention features from records and compared these to current clinical status. We performed similar assessments on a cohort of patients with other convulsive epilepsies (OCE) and compared outcomes of the two groups.

Participants

Participants were patients with World Health Organization defined NS and patients with OCE attending the same treatment centres.

Outcome measures

The primary outcome was the proportion of patients with seizure freedom (≥1 month without seizures). Secondary outcome measures included reduction in seizure frequency, resolution of behaviour and emotional difficulties, independence in basic self care and return to school.

Results

Among 484 patients with NS and 476 with OCE, the intervention resulted in marked improvements; compared to the pre-intervention state, 121/484(25.0%) patients with NS achieved seizure freedom and there was >70% reduction in seizure frequency; behaviour and emotional difficulties resolved in 194/327(59%); 193/484(40%) had enrolled in school including 17.7% who had earlier withdrawn due to severe seizures and over 80% had achieved independence in basic self care. These improvements were however less than that in patients with OCE of who, 243/476(51.1%) were seizure free and the seizure frequency had reduced by 86%.

Conclusions

Ugandan children with NS show substantial clinical and functional improvements with symptomatic treatments suggesting that NS is probably a treatable disorder. Uncontrolled seizures may be a major contributor to the neuro-cognitive decline in this syndrome.

For peer review only - http://bmjopen.bmj.com/site/about/guidelines.xhtml

Treatment Outcomes of Nodding Syndrome in Uganda

ARTICLE SUMMARY

Article focus

- This paper examines the clinical and functional outcomes of a symptomatic treatment intervention for children of nodding syndrome in Uganda, and compares these outcomes to that of patients with other convulsive epilepsies in the same setting.

Key messages

- The symptoms and psychomotor functioning of patients with nodding syndrome improve with symptomatic treatments suggesting that nodding syndrome is probably a reversible epileptic encephalopathy. Symptom reversibility may depend on timing of interventions.
- The improvements are however less than that observed in patients with other convulsive epilepsies suggesting that seizures in nodding syndrome may be less anticonvulsant sensitive compared to seizures in the other convulsive epilepsies.

Strengths and limitations of the study

- This is the largest cohort of nodding syndrome ever reported on to date. The report examines pre-intervention clinical and functional features before a well designed treatment intervention was implemented and how these improve over the course of the ensuing year. The outcomes are also compared to that of a similar cohort with other epilepsies.
- However, we did not conduct a prospective study but rather before and after cross sectional studies meaning that we cannot comment on the incidence of death or on loss to follow. In addition, we relied on patient records for the pre-intervention features. Other than head nodding, seizures in nodding syndrome are similar to seizures in other convulsive epilepsies and over time, head nodding may cease in some patients with nodding syndrome increasing the risk of misclassification. In addition, we did not determine compliance to antiepileptic drugs or have reports of adverse effects patients experienced while on treatment and did not have a detailed documentation of the nutritional and cognitive stimulatory treatments each child received. We however limited the effects of such bias by choosing only a few outcome measures that are not easily confused.

For peer review only

Treatment Outcomes of Nodding Syndrome in Uganda

BACKGROUND

Nodding syndrome is a poorly understood devastating neurologic disorder affecting several thousand children in the sub-Saharan African countries of South Sudan, Uganda and Tanzania.[1-6] The syndrome is characterised by almost daily atonic seizures manifesting as clusters of head nods[7] and complicated by tonic clonic, focal, myoclonic and/or atypical absence seizures, cognitive and motor decline, malnutrition, behavioural and emotional difficulties[2, 8]. The aetiology is unknown although the syndrome has been associated with infestation with *Onchocerca volvulus.*[2, 9, 10] Studies of Tanzanian and Ugandan patients have concluded that nodding syndrome is probably symptomatic generalised epilepsy.[2, 7, 8]

In Uganda, a multidisciplinary team developed management guidelines for care[11]. The objective was to relieve symptoms, offer primary and secondary prevention of disability, and rehabilitation to improve function. The most important clinical needs were identified as seizure control, relief of behavioural and emotional difficulties, nutritional, physical and cognitive rehabilitation. The first group of patients were enrolled in March 2012. We evaluated clinical outcomes of this intervention after a minimum of 12 months. We hypothesised that if treated with appropriate anticonvulsants, patients with nodding syndrome would achieve similar seizure control like patients with other convulsive epilepsies. We therefore in addition, compared outcomes of patients with nodding syndrome to patients with other convulsive epilepsies.

For peer review only - http://bmjopen.bmj.com/site/about/guidelines.xhtml

Treatment Outcomes of Nodding Syndrome in Uganda

METHODS

Design and setting

This was a cross sectional survey of a cohort of patients with nodding syndrome that evaluated the clinical and functional outcomes of patients receiving the Ugandan Ministry of Health treatment intervention at least 12 months after initiation of therapy. We performed a similar evaluation on a cohort of patients with other convulsive epilepsies that attended the same centres and compared the two groups. The study was conducted in northern Uganda, the region most affected by nodding syndrome in the country. This region also suffered a protracted armed rebellion that lasted over 20 years[12] resulting into massive internal displacement. It is only in the past 6-7 years that peace prevailed and the population returned to their homes.

Participants

Participants were patients with either nodding syndrome or other convulsive epilepsies receiving treatment at any one of the nodding syndrome treatment centres in the seven districts of Lamwo, Kitgum, Pader, Gulu, Amuru, Lira and Oyam. The definition of head nodding and diagnosis of nodding syndrome is in accord with the criteria developed by international consensus during the World Health Organization facilitated meeting on Nodding Syndrome in Kampala, 2012[13]. Head nodding was defined as repetitive, involuntary drops of the head on to the chest in previously normal persons. We included probable and confirmed cases only. Children with other convulsive epilepsies were those with active (at least one in the past year) tonic-clonic or focal jerking epileptic seizures. Those with onset of symptoms outside of the ages 3-18 years were excluded to allow comparability with nodding syndrome patients.

The intervention

The nodding syndrome treatment centres in Lamwo, Kitgum and Pader were opened in March 2012 followed by those in Amuru, Gulu, Lira and Oyam in June 2012. Prior to this, clinicians and nurses at each centre underwent a five-day training on the management of nodding syndrome using the specified guideline[14]. The training which also included general principles of epilepsy treatment was provided through didactic lectures, role play, bedside clinical teachings and demonstrations by the same team that developed the guidelines. At the end of the five days, each team returned to their centre and worked with the trainers to initiate provision of care. Other than the centre in Kitgum which is a district hospital (a level V health centre), all the others were health centre III. At each centre, clinical service was led by a medical or psychiatric clinical officer (individuals with a diploma in clinical medicine or psychiatry after three years of training), general

For peer review only - http://bmjopen.bmj.com/site/about/guidelines.xhtml

and psychiatric nurses, laboratory technicians and either a physiotherapist or occupational therapist. In Kitgum hospital, the team was led by a medical officer (MBChB). These teams were supported by local lay volunteers - village health workers – who coordinated follow up and ambulatory care in homes. In each district, supervisory oversight was provided by a district nodding syndrome focal person, the District Health Officer and the district nodding syndrome committee while nationally, there was a national nodding syndrome coordinator who brought everyone together. Over the next 12 months, each centre received support supervision visits on at least two occasions to maintain skills and attend to issues arising.

Details of the treatment are described elsewhere[11]. In summary, inpatient emergency care was offered to patients with life threatening co-morbidities. Ambulatory and community care was offered to patients without co-morbidities or those with non-life threatening co-morbidities. Sodium valproate was the first-line anticonvulsant starting at 10mg/kg/day in two divided doses and the dose titrated to a maximum of 40mg/kg/day. The patient's family was provided with supplemental food rations every 2-4 weeks. Severely malnourished patients with medical complications were treated as inpatients and those with uncomplicated severe malnutrition were treated as outpatients with ready to use therapeutic feeds. This was provided as Plumpy'Nut® a product of Nutriset, (Normandy, France). Plumpy'Nut is made of peanut paste, vegetable oil, powdered milk and sugar, vitamins (A, B-complex, C, D, E and K) and minerals (calcium, phosphorus, potassium, magnesium, zinc, copper, iron, iodine, sodium and selenium) all combined in a foil pouch. Each 92g pack provides 500 calories. Management of behaviour and emotional difficulties included counselling and referral of those with severe symptoms to mental health services. Other management included physical, speech and language therapy and cognitive stimulation.

Children with other convulsive epilepsies were provided with first-line anticonvulsants (carbamazepine, phenobarbitone, phenytoin or sodium valproate) or continued to receive earlier prescribed anticonvulsants but the dose adjusted appropriately. A new anticonvulsant was introduced if an inappropriate drug was being provided. Anticonvulsants such as oxcarbazepine, lamotrigine, levetiracetam, and topiramate are unavailable in the public health service in Uganda. Families of patients with other convulsive epilepsies were also provided with similar supplemental feeding. In addition, parents/carers of both groups of patients were educated on seizures, epilepsy, adherence to antiepileptic drugs and prevention of seizure related injuries.

For peer review only - http://bmjopen.bmj.com/site/about/guidelines.xhtml

Treatment Outcomes of Nodding Syndrome in Uganda

Sample size

In a preliminary evaluation of the treatment outcomes of nodding syndrome after seven months of intervention, we documented (from parental or carer report) that 5/47 (10%) had achieved seizure freedom (no head nodding or convulsive seizures) for at least 30 days prior to the visit. Using these findings, we estimated that a sample of 432 patients will be sufficient at 5% level of significance and 90% power to detect a 10% increase in this proportion to 20% after 12 months of treatment. Secondly, up to 70% of children with new onset convulsive epilepsies achieve terminal seizure remission with drug treatment.[15, 16] The onset of seizure remission is often evident within the first year of treatment[15]. Using these findings, we estimated that with a sample of 461 nodding syndrome patients and a similar number with other convulsive epilepsies, we will be able to, at 90% power and 5% level of significance, reject the null hypothesis that there is no difference in the proportions of patients with nodding syndrome or other convulsive epilepsies achieving seizure freedom with 12 months therapy. We set to recruit the larger sample.

Study procedures and measurements

As of 30 June 2013, there were a reported 3,295 patients with probable or confirmed nodding syndrome receiving care at the seven centres. We used proportionate sampling to estimate the number of participants to be recruited from each centre and consecutively recruited patients as they presented until the sample was achieved. Data was collected between 1st July 2013 and 30th September 2013. One of two investigators (RI or BTO) first conducted a day's training on the study procedures followed by a joint clinic with the clinicians at the centre. The local clinical team subsequently worked independently until study completion. Case record forms were completed from data abstracted from pre-intervention records, direct inquiry from parents/carers and on physical exam. The pre-intervention seizure burden, weight and height, and behaviour or emotional difficulty was obtained from records. We defined seizures as head nodding or convulsive seizures and defined seizure burden as the number of clusters of head nodding and/or convulsive seizures per unit time.

In the clinic, parents/carers reported on current seizures, behaviour and emotional difficulties. The weight was measured using a stand on electronic scale while height or length was measured using a stadiometer. Independence in basic self care (self feeding, dressing and using a toilet), the status of schooling, and ability to appropriately help with culturally and age-appropriate homecare activities (e.g. sweeping the compound) were obtained from the parents or carers. The parents and carers were also asked to provide an overall assessment of improvements or

For peer review only - http://bmjopen.bmj.com/site/about/guidelines.xhtml

worsening of symptoms over the year on an ordinal scale (markedly improved, some improvement, no improvement or worse).

Outcome measures

The primary outcome was the proportion of patients who had achieved seizure freedom (defined as ≥1 month without seizures [no head nodding and/or convulsive seizures observed by the parent/carer prior to follow up visit]). Secondary outcomes included reduction in seizure burden (reduction in the mean number of clusters of head nods and/or convulsive seizures per unit time), the proportions of patients with independence in basic self care, resolution of behaviour and emotional difficulties, and enrolment in school.

Data management and statistical analysis

Data was collected on case record forms and double-entered into a Microsoft Access 2007 database. Data analysis was performed using STATA version 12.0 (STATA Corp, Tx). The two patient groups were considered as two independent single samples and paired data (before initiation of therapy and at least 12 months later) analysis was performed for each group. Thus, we determined the proportions of patients with nodding syndrome with seizure freedom before and after 12 months and the proportions with the different secondary outcomes. A one sample t-test was used to compare means of normally distributed continuous data, the Mann-Whitney U test for medians of skewed data and McNemar's test for categorical data. The outcomes of patients with nodding syndrome were then compared to those of patients with other convulsive epilepsies. We then examined for variables associated with seizure freedom and performed a logistic regression analysis to determine variables independently associated with achieving seizure freedom.

For peer review only - http://bmjopen.bmj.com/site/about/guidelines.xhtml

Treatment Outcomes of Nodding Syndrome in Uganda

RESULTS

General descriptions

A total of 1,322 subjects were screened in six out of the seven districts. Oyam district, which had only eight patients with nodding syndrome, was not visited. Two hundred and fifteen subjects were ineligible. Another 147 were also excluded for different reasons. Thus, 960 participants (484 with nodding syndrome and 476 with other convulsive epilepsies) were available for the study (**figure 1**).

The two groups were of similar age and gender; the mean (SD) age of patients with nodding syndrome was 13.7(3.6) years and that for patients with other convulsive epilepsies was 13.0(2.9) years, p=0.998; 281/484 (58.1%) subjects with nodding syndrome and 267/476 (56.1%) with other convulsive epilepsies were male, p=0.538. However, participants with nodding syndrome had experienced a longer duration of symptoms (median 5[IQR 3, 6] years) compared to patients with other epilepsies, (median 4 [IQR 2, 6] years), p<0.001.

The median daily dose of sodium valproate in patients with nodding syndrome was 16(IQR 12, 21) mg/kg/day with most (298/484, 61.6%) on relatively low doses (<20mg/kg/day). The majority of the patients with other convulsive epilepsies (421/476, 88.5%) were on carbamazepine, phenobarbitone or phenytoin monotherapy. The remaining 55 were either on sodium valproate (40/476, 8.4%) or combinations of the above anticonvulsants (15/476, 3.1%).

Outcomes of interventions

a) Seizures

There was marked reduction in seizures with the intervention; overall, 25% (95% CI 21, 29) of nodding syndrome patients achieved seizure freedom. Both the frequency head nodding and of convulsive seizures reduced by over 70%. The reduction in seizure burden was even more marked in patients with other convulsive epilepsies; 51% (95% CI 46.4, 55.6) achieved seizure freedom and the overall burden of seizures decreased by 86%, **(table 1)**.

Although the effects of sodium valproate on seizure control in nodding syndrome was evident at relatively low doses, additional patients achieved seizure freedom with dose escalation. Thus, 87/298 (29.2%) patients were seizure free on sodium valproate <20mg/kg/day and an additional 34/186 (18.3%) achieved seizure freedom with dose increases to 20-40mg/kg/day.

For peer review only - http://bmjopen.bmj.com/site/about/guidelines.xhtml

Treatment Outcomes of Nodding Syndrome in Uganda

Table 1 Pre-interventions features and features at least 12 months after initiation of a symptomatic treatment intervention in patients with nodding syndrome or other convulsive epilepsies

	Patients with nodding syndrome, N=484			Other convulsive epilepsies, N=476		
	Pre- intervention status	Features ≥ 12 months later	P value	Pre-intervention status	Features ≥ 12 months later	P value
**Patients with seizure freedom*, %**	8 (2%) [95% CI 0.07, 3.2]	121 (25.0%) [95% CI 21.2, 29.1]	<0.001	8 (2%) [95% CI 0.7, 3.3]	243 (51.1%) [95% CI 46.4, 55.6]	<0.001
**Daily clusters of head nods, median (IQR)**	4 (IQR 3, 6)	1 (IQR 0, 2)	<0.001	-	-	-
**Patients with behaviour & emotional difficulties, %**	327/484 (67.6%) [95% CI 63.2, 71.7]	133 (27.5%) [95% CI 23.5, 33.7]	<0.001	250/476 (52.5%) [95% CI 47.9, 57.1]	105 (22.1%) [95% CI 18.4, 26.1]	<0.001
**GMFCS score****						
**1**	185/282 (64.0%)	223/282 (79.1%)	<0.001***	212/288 (73.6%)	239/288 (83.0%)	<0.001***
**2**	58/282 (20.1%)	39/282 (13.8%)		41/288 (14.1%)	39/288 (13.5%)	
**3**	39/288 (13.5%)	39/288 (13.5%)		39/288 (13.5%)	10/288 (3.5%)	
**4 and 5**	39/288 (13.5%)	0 (0)		2/288 (0.7%)	0 (0)	
**Independence in basic self care, %**	174 (36.0%) [95% CI 31.7, 40.4]	402 (83.1%) [95% CI 79.4, 86.3]	< 0.001	206 (43.3%) [95% CI 38.8, 47.9]	397 (83.4%) [95% CI 79.8, 86.6]	<0.001
**Able and performs culturally and age appropriate homecare activities, %**	152 (31.4%) [95% CI 27.2, 37.4]	372 (76.9%) [95% CI 72.8, 80.5]	<0.001	187 (39.3%) [95% CI 34.9, 43.8]	382 (80.3%) [95% CI 76.4, 83.7]	<0.001
**Enrolled at and attending school, %**	107 (22.1%) [95% CI 18.5, 26.1]	193 (39.9%) [95% CI 35.5, 44.4]	<0.001	170 (35.7%) [95% CI 31.4, 40.2]	250 (52.5%) [95% CI 47.9, 57.1]	<0.001

*≥1 month without seizures
**GMFCS=Gross Motor Function Classification Score; N=282; i.e. Only 282 patients with nodding syndrome had paired GMFCS pre and post interventions scores obtained.
***Chi square test for trend with Yate's correction

We repeated diagnostic electroencephalogram (EEG) recordings for three patients with nodding syndrome who were part of the 22 we reported on earlier[8]. The recordings showed clear improvements in background EEG and reductions in previously widespread interictal epileptiform discharges. All three were on sodium valproate 20-25mg/kg/day and were experiencing only occasional convulsive seizures but no head nodding.

For peer review only - http://bmjopen.bmj.com/site/about/guidelines.xhtml

Treatment Outcomes of Nodding Syndrome in Uganda

b) Behaviour and emotional difficulties

Behaviour and emotional difficulties were reported in 327(67.6%) participants with nodding syndrome and in 250(52.5%) with other convulsive epilepsies prior to the intervention. Among participants with nodding syndrome, these included aggressive and destructive behaviour (186/484, 39.5%), wandering or running away (113/484, 23.4%) and periods of low mood (114/484, 23.6%). Over the 12 months, the difficulties resolved in 194/327 (59.3%) nodding syndrome and in 145/250 (58.0%) patients with other convulsive epilepsies. Improvements were most evident in nodding syndrome patients initially reporting wandering, aggressive and destructive behaviour. Psychotropic drugs (haloperidol) were prescribed for only three patients with severe difficulties and two received anxiolytic drugs. An additional 62(12.8%) nodding syndrome patients, especially those with uncontrolled or worsening seizures, developed new onset behaviour and emotional difficulties; these included 44 (9.1%) aggressive and destructive behaviour, 18(3.7%) wandering, and 21(4.3%) mood problems. Wandering behaviour was uncommon among patients with other convulsive epilepsies in whom impulsive behaviour and hyperactivity were more common.

c) Independence in basic self care

Prior to the intervention, 174/484(36.0%) patients with nodding syndrome were independent in basic self care. This proportion had increased to 402/484(83.1%) by the time of the survey, $p<0.001$. Similar improvements were observed in patients with other convulsive epilepsies. Thus, 397/476(83.4%) of these patients were independent in basic self care at the time of the survey up from 270/476 (56.7%) prior to intervention, $p<0.001$.

d) School of attendance

A total of 443 patients (193/484, 39.9% with nodding syndrome and 250/476, 52.5% with other convulsive epilepsies) were enrolled in and attending school at the time of the survey. This included 86/484 (17.8%) patients with nodding syndrome and 80/476 (16.8%) patients with other convulsive epilepsies who had returned to school with seizure control and improvements in other symptoms. Although these children have returned to school, parents reported that 90/193(46.6%) patients with nodding and 76/250(30.4%) patients with other epilepsies still performing poorly in school.

For peer review only - http://bmjopen.bmj.com/site/about/guidelines.xhtml

Treatment Outcomes of Nodding Syndrome in Uganda

e) **Qualitative assessment of improvement by parents and carers**

On an ordinal subjective scale, parents felt that 112/484(23.1%) patients with nodding syndrome and 253/476(53.2%) patients with other convulsive epilepsies had improved markedly. Another 325/484(67.2%) patients with nodding syndrome and 194/476(40.8%) with other convulsive epilepsies had some improvement. The number of patients with nodding syndrome who could participate and help their parents with home care tasks increased from 152/484 (31.4%) to 372/484 (76.9%) with the intervention. Only 47/484(9.7%) patients with nodding syndrome and 29/476(6.1%) with other convulsive epilepsies had no improvement in symptoms or became worse over the period of intervention.

Prognostic factors for seizure freedom

We examined the relationship between gender, age at onset of symptoms, duration of symptoms, baseline seizure frequency, presence of behaviour and emotional difficulties, whether the child had head nodding only or head nodding plus (other seizures), antiepileptic drug dose and achieving seizure freedom. Only a lower number of clusters of head nods prior to the intervention (adjusted OR 0.80 [95% CI 0.72-0.88], $p<0.001$) and response to a lower antiepileptic drug dose (adjusted OR 0.96 (95% CI 0.93, 0.99], p=0.046) were independently associated with achieving seizure freedom.

DISCUSSION

Our study aimed to determine the clinical outcomes and therefore, the effectiveness of a symptomatic treatment intervention for nodding syndrome. We documented substantial clinical and functional improvements with the intervention. The findings suggest that nodding syndrome is probably a reversible encephalopathy. The improvements we observed were however less than that seen in patients with other convulsive epilepsies suggesting that epileptic seizures in nodding syndrome may be less anticonvulsant sensitive compared to seizures in the other convulsive epilepsies.

Although the number of patients who achieved seizure freedom was modest, our findings suggest that a treatment package of selected anticonvulsants, psycho-behavioural interventions and nutritional and physical rehabilitation can control seizures, improve function and even reverse some severe functional disability in nodding syndrome. This observation seems to concur with a report from Tanzania where symptoms of nodding syndrome completely resolved in four of the original cohort of 62 patients.[17] Even though we did not perform specific cognitive testing or brain

For peer review only - http://bmjopen.bmj.com/site/about/guidelines.xhtml

Treatment Outcomes of Nodding Syndrome in Uganda

imaging to objectively document functional and structural improvements with the intervention, comparisons of a pre-intervention and repeat EEG recordings in three patients with previous recordings demonstrated clear improvements in background EEG and reductions in the previously widespread interictal epileptiform discharges.[8]

Clinical trials comparing treatment of seizures in nodding syndrome with sodium valproate to treatment with other anticonvulsants such as lamotrigine or levetiracetam either as monotherapy or as add on therapy may be considered. In addition and especially for patients whose symptoms are either not controlled or became worse on therapy, other strategies may be considered. Epileptic encephalopathy is a possibility especially in patients with severe and persistent symptoms. Can therapy with benzodiazepines, high dose steroids or other immunosuppressant drugs be considered?[18]

The aetiology of nodding syndrome is still unknown. In all three countries where nodding syndrome has been described, it has been associated with infestation by *Onchocerca volvulus*.[2, 9, 19] Uganda is in its second year of twice yearly mass administration of ivermectin (an antimicrofilarial agent active only against the mirofilaria but not the adult parasite). Other strategies that target the both microfiliaria and the adult worms and/or their co-symbiotic bacteria, Wolbachiae, may be considered as potential specific therapy.[20, 21]
Despite these improvements, parents reported that the majority of the 40% children who returned to school continued to perform poorly. There is need to examine whether the continued poor academic performance is due to irreparable brain injury or an underlying ongoing aetio-pathogenic process. To date, there are no systematic studies of cognitive function in nodding syndrome. Such studies will help define areas of functional deficits and document improvements preferably using tools that can be applied across different regions with minimal modification to allow comparison.

We did not apply specific psychiatric diagnostic tools to patients with behaviour and emotional difficulties to be able to make distinct psychiatric diagnoses. A few children with severe difficulties were attended to by the local mental health services and some given psychotropic drugs. The majority of the 194 children in whom behaviour and emotional difficulties resolved however improved without psychotropic drugs but with seizure control suggesting that in nodding syndrome, some of these features may be co-morbidities of epilepsy. Wandering behaviour may be an ictal event[8]. Some patients may also have benefitted from the effects of sodium valproate

For peer review only - http://bmjopen.bmj.com/site/about/guidelines.xhtml

on behaviour; in a recent case series of Ugandan children, Musisi et al documented improvements in some patients receiving antidepressants.[22] Put together, these findings suggest that psychotropic drugs may be considered for some patients with nodding syndrome especially those with severe symptoms.

Study limitations

First, other than head nodding, seizures in nodding syndrome may be similar to seizures in other convulsive epilepsies and over time, head nodding may cease in some patients.[17] This scenario opens room for potential misclassification of disease as the current disease criteria heavily leans on clinical observations. Secondly, we did not perform a prospective study; instead, we relied on patient records for pre-intervention features. Third, we did not determine compliance to antiepileptic drugs or have reports of adverse effects patients experienced while on treatment. We also did not have a detailed documentation of the nutritional therapy and the cognitive stimulatory activities each child received and did not assess the effect of home environment on outcome. We however limited the effects of such bias by choosing only a few and fairly robust outcome measures.

Failure to conduct a prospective study means that we cannot comment on the incidence of death or on patients who might have discontinued follow up care (e.g. due to deterioration in symptoms, severe motor disability or loss of faith in the treatment) leading to an over estimate of the effect. Such an effect if any is likely minimal. From Ministry of Health epidemiological surveillance reports, only 12 patients with probable nodding syndrome died over the period of observation mostly from seizure related events.

Conclusions

The symptoms and psychomotor functioning of patients with nodding syndrome improve with symptomatic treatments suggesting that nodding syndrome is probably a reversible epileptic encephalopathy. Symptom reversibility may depend on the timing of interventions. Uncontrolled epileptic seizures may be a major contributor to the neuro-cognitive decline and disability in this syndrome. Further studies to elucidate these findings recommended.

For peer review only

For peer review only - http://bmjopen.bmj.com/site/about/guidelines.xhtml

Treatment Outcomes of Nodding Syndrome in Uganda

Author's contributions

RI, HN, CA, BBM, AKM, ROO, AKM, ADM and BTO designed the intervention and the study and participated in supervising patient care. RI and HN performed the data analysis. RI wrote the first draft and all participated in data interpretation and provided a critical review of the manuscript.

Funding

The study was funded by the Government of Uganda. Dr Idro is partly supported by the Wellcome Trust through a Director's discretionary research funds. The funding agency had no role in data collection, analysis and interpretation of results and the decision to submit for publication.

Competing interests

There are no conflicts of interest to report.

Ethics approval

Makerere University School of Medicine Research and Ethics Committee

Data sharing statement

No additional data are available.

Acknowledgements

We would like the thank Local District Health Services in Lamwo, Kitgum, Pader, Amuru, Gulu, and Lira who made the study possible, Keron Ssebyala who managed the data and Prof Gretchen Birbeck, who critically reviewed the first draft of the this manuscript and offered helpful comments to improve it.

For peer review only

Treatment Outcomes of Nodding Syndrome in Uganda

Reference

1. Lacey M. Nodding disease: mystery of southern Sudan. Lancet neurology. 2003; 2(12): 714.
2. Winkler AS, Friedrich K, Konig R, Meindl M, Helbok R, Unterberger I, et al. The head nodding syndrome--clinical classification and possible causes. Epilepsia. 2008; 49(12): 2008-15.
3. Kaiser C, Pion S, Boussinesq M. Head nodding syndrome and river blindness: a parasitologic perspective. Epilepsia. 2009; 50(10): 2325-6.
4. Edwards J. Nodding disease confounds clinicians. CMAJ : Canadian Medical Association journal = journal de l'Association medicale canadienne. 2012; 184(11): E585-6.
5. Korevaar DA, Visser BJ. Reviewing the evidence on nodding syndrome, a mysterious tropical disorder. International journal of infectious diseases : IJID : official publication of the International Society for Infectious Diseases. 2013; 17(3): e149-52.
6. Dowell SF, Sejvar JJ, Riek L, Vandemaele KA, Lamunu M, Kuesel AC, et al. Nodding syndrome. Emerging infectious diseases. 2013; 19(9): 1374-84.
7. Sejvar JJ, Kakooza AM, Foltz JL, Makumbi I, Atai-Omoruto AD, Malimbo M, et al. Clinical, neurological, and electrophysiological features of nodding syndrome in Kitgum, Uganda: an observational case series. Lancet neurology. 2013; 12(2): 166-74.
8. Idro R, Opoka RO, Aanyu HT, Kakooza-Mwesige A, Piloya-Were T, Namusoke H, et al. Nodding syndrome in Ugandan children--clinical features, brain imaging and complications: a case series. BMJ open. 2013; 3(5).
9. Tumwine JK, Vandemaele K, Chungong S, Richer M, Anker M, Ayana Y, et al. Clinical and epidemiologic characteristics of nodding syndrome in Mundri County, southern Sudan. African health sciences. 2012; 12(3): 242-8.
10. Foltz JL, Makumbi I, Sejvar JJ, Malimbo M, Ndyomugyenyi R, Atai-Omoruto AD, et al. An Epidemiologic Investigation of Potential Risk Factors for Nodding Syndrome in Kitgum District, Uganda. PloS one. 2013; 8(6): e66419.
11. Idro R, Musubire KA, Byamah Mutamba B, Namusoke H, Muron J, Abbo C, et al. Proposed guidelines for the management of nodding syndrome. Afr Health Sci. 2013; 13(2): 219-32.
12. Wendo C. Northern Uganda humanitarian crisis shocks UN chief. Rebels in northern districts have left people trapped in hunger, disease, poverty, and fear. Lancet. 2003; 362(9398): 1818.
13. WHO. International Scientific Meeting on Nodding Syndrome; 2012.
14. Idro R, Musubire KA, Byamah Mutamba B, Namusoke H, Muron J, Abbo C, et al. Proposed guidelines for the management of nodding syndrome. African health sciences. 2013; 13(2): 219-32.
15. Hauser E, Freilinger M, Seidl R, Groh C. Prognosis of childhood epilepsy in newly referred patients. Journal of child neurology. 1996; 11(3): 201-4.
16. Berg AT, Shinnar S, Levy SR, Testa FM, Smith-Rapaport S, Beckerman B, et al. Two-year remission and subsequent relapse in children with newly diagnosed epilepsy. Epilepsia. 2001; 42(12): 1553-62.
17. Winkler AS, Wallner B, Friedrich K, Pfausler B, Unterberger I, Matuja W, et al. A longitudinal study on nodding syndrome--a new African epilepsy disorder. Epilepsia. 2014; 55(1): 86-93.
18. Parisi P, Spalice A, Nicita F, Papetti L, Ursitti F, Verrotti A, et al. "Epileptic encephalopathy" of infancy and childhood: electro-clinical pictures and recent understandings. Current neuropharmacology. 2010; 8(4): 409-21.
19. Nodding syndrome - South Sudan, 2011. MMWR Morb Mortal Wkly Rep. 2012; 61(3): 52-4.
20. Hoerauf A, Specht S, Buttner M, Pfarr K, Mand S, Fimmers R, et al. Wolbachia endobacteria depletion by doxycycline as antifilarial therapy has macrofilaricidal activity in onchocerciasis: a randomized placebo-controlled study. Medical microbiology and immunology. 2008; 197(3): 295-311.

For peer review only - http://bmjopen.bmj.com/site/about/guidelines.xhtml

21. **Hoerauf A, Mand S, Adjei O, Fleischer B, Buttner DW. Depletion of wolbachia endobacteria in Onchocerca volvulus by doxycycline and microfilaridermia after ivermectin treatment. Lancet. 2001; 357(9266): 1415-6.**

22. **Musisi S, Akena D, Nakimuli-Mpungu E, Abbo C, Okello J. Neuropsychiatric perspectives on nodding syndrome in northern Uganda: a case series study and a review of the literature. African health sciences. 2013; 13(2): 205-18.**

For peer review only

Figure 1 Subject recruitment

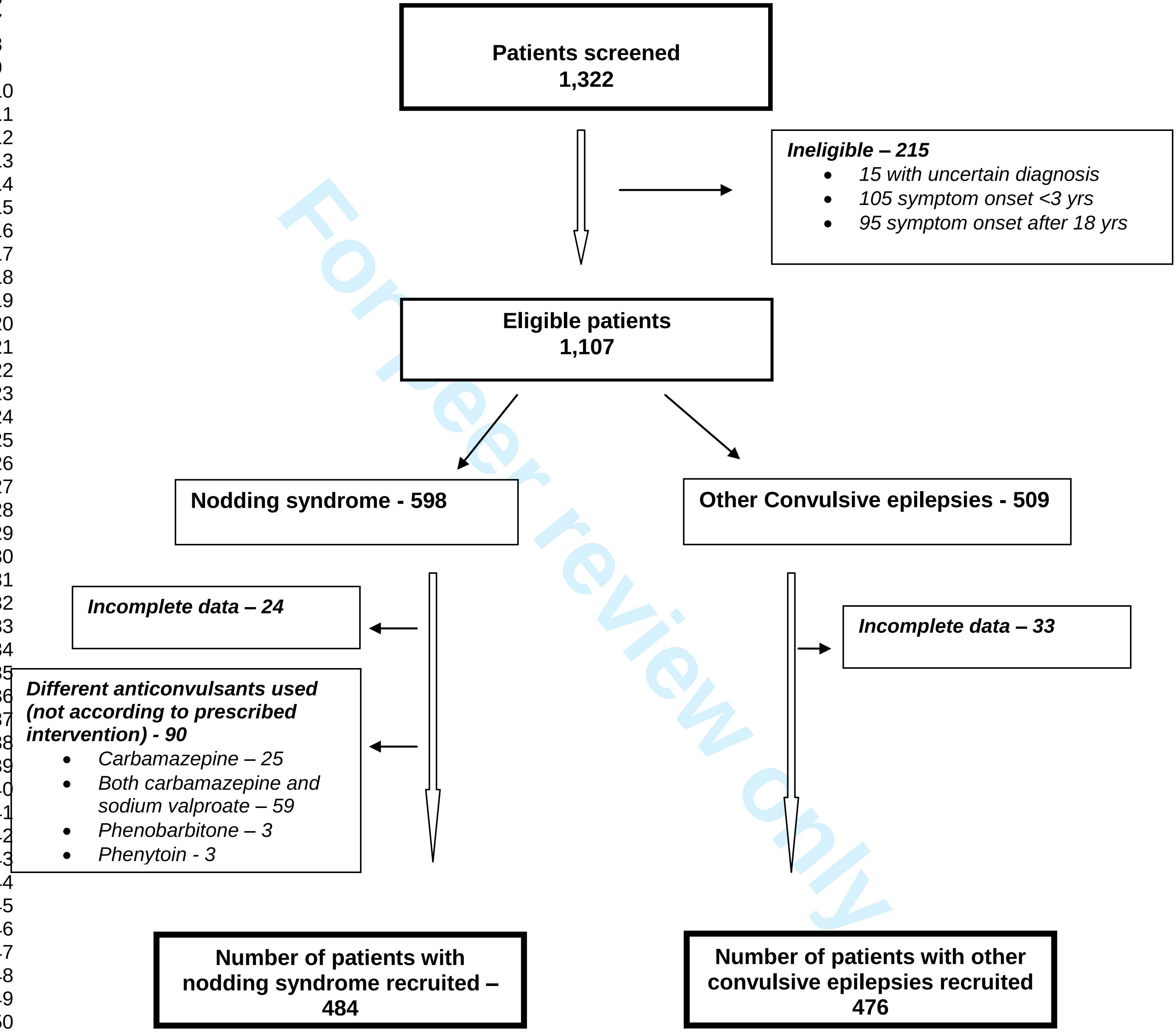

For peer review only

STROBE 2007 (v4) Statement—Checklist of items that should be included in reports of *cross-sectional studies*

Section/Topic	Item #	Recommendation	Reported on page #
**Title and abstract**	1	(*a*) Indicate the study's design with a commonly used term in the title or the abstract	1
		(*b*) Provide in the abstract an informative and balanced summary of what was done and what was found	2
**Introduction**			
Background/rationale	2	Explain the scientific background and rationale for the investigation being reported	4
Objectives	3	State specific objectives, including any prespecified hypotheses	4
**Methods**			
Study design	4	Present key elements of study design early in the paper	5
Setting	5	Describe the setting, locations, and relevant dates, including periods of recruitment, exposure, follow-up, and data collection	5-7
Participants	6	(*a*) Give the eligibility criteria, and the sources and methods of selection of participants	5-6
Variables	7	Clearly define all outcomes, exposures, predictors, potential confounders, and effect modifiers. Give diagnostic criteria, if applicable	5, 7, 8
Data sources/ measurement	8*	For each variable of interest, give sources of data and details of methods of assessment (measurement). Describe comparability of assessment methods if there is more than one group	6-7
Bias	9	Describe any efforts to address potential sources of bias	*6*
Study size	10	Explain how the study size was arrived at	7
Quantitative variables	11	Explain how quantitative variables were handled in the analyses. If applicable, describe which groupings were chosen and why	8
Statistical methods	12	(*a*) Describe all statistical methods, including those used to control for confounding	8
		(*b*) Describe any methods used to examine subgroups and interactions	N/A
		(*c*) Explain how missing data were addressed	Figure 1
		(*d*) If applicable, describe analytical methods taking account of sampling strategy	8
		(*e*) Describe any sensitivity analyses	N/A
**Results**			

For peer review only

Participants	13*	(a) Report numbers of individuals at each stage of study—eg numbers potentially eligible, examined for eligibility, confirmed eligible, included in the study, completing follow-up, and analysed	9, Figure 1
		(b) Give reasons for non-participation at each stage	9, Figure 1
		(c) Consider use of a flow diagram	Figure 1
Descriptive data	14*	(a) Give characteristics of study participants (eg demographic, clinical, social) and information on exposures and potential confounders	9
		(b) Indicate number of participants with missing data for each variable of interest	Figure 1, table 1
Outcome data	15*	Report numbers of outcome events or summary measures	9-12, table 1
Main results	16	(*a*) Give unadjusted estimates and, if applicable, confounder-adjusted estimates and their precision (eg, 95% confidence interval). Make clear which confounders were adjusted for and why they were included	11, Table 1,
		(*b*) Report category boundaries when continuous variables were categorized	9-12, table 1
		(*c*) If relevant, consider translating estimates of relative risk into absolute risk for a meaningful time period	N/A
Other analyses	17	Report other analyses done—eg analyses of subgroups and interactions, and sensitivity analyses	N/A
**Discussion**			
Key results	18	Summarise key results with reference to study objectives	12
Limitations	19	Discuss limitations of the study, taking into account sources of potential bias or imprecision. Discuss both direction and magnitude of any potential bias	14
Interpretation	20	Give a cautious overall interpretation of results considering objectives, limitations, multiplicity of analyses, results from similar studies, and other relevant evidence	12-13
Generalisability	21	Discuss the generalisability (external validity) of the study results	12, 14
**Other information**			
Funding	22	Give the source of funding and the role of the funders for the present study and, if applicable, for the original study on which the present article is based	15

*Give information separately for cases and controls in case-control studies and, if applicable, for exposed and unexposed groups in cohort and cross-sectional studies.

Note: An Explanation and Elaboration article discusses each checklist item and gives methodological background and published examples of transparent reporting. The STROBE checklist is best used in conjunction with this article (freely available on the Web sites of PLoS Medicine at http://www.plosmedicine.org/, Annals of Internal Medicine at http://www.annals.org/, and Epidemiology at http://www.epidem.com/). Information on the STROBE Initiative is available at www.strobe-statement.org.

For peer review only

BMJ Open

Nodding syndrome patients in Uganda improve with symptomatic treatment; a cross sectional study

Journal:	*BMJ Open*
Manuscript ID:	bmjopen-2014-006476.R1
Article Type:	Research
Date Submitted by the Author:	01-Oct-2014
Complete List of Authors:	Idro, Richard; Mulago Hospital/Makerere University, Department of Paediatrics and Child Health; University of Oxford, Nuffield Department of Medicine, Centre for Tropical Medicine and Global Health<br>Namusoke, Hanifa; Mulago Hospital/Makerere University, Department of Paediatrics and Child Health<br>Abbo, Catherine; Mulago Hospital/Makerere University, Department of Psychiatry<br>Mutamba, Brian; Mulago Hospital/Makerere University, Department of Psychiatry; Butabika Hospital, Department of Psychiatry<br>Kakooza, Angelina; Mulago Hospital/Makerere University, Department of Paediatrics and Child Health<br>Opoka, Robert; Mulago Hospital/Makerere University, Department of Paediatrics and Child Health<br>Musubire, Abdu; Mulago Hospital/Makerere University, Department of Internal Medicine<br>Mwaka, Amos; Mulago Hospital/Makerere University, Department of Internal Medicine<br>Opar, Bernard; Ministry of Health Headquarters, Department of Clinical Services
**Primary Subject Heading**:	Neurology
Secondary Subject Heading:	Global health
Keywords:	Paediatric neurology < PAEDIATRICS, Epilepsy < NEUROLOGY, Developmental neurology & neurodisability < PAEDIATRICS

SCHOLARONE™ Manuscripts

Treatment Outcomes of Nodding Syndrome in Uganda

Nodding syndrome patients in Uganda improve with symptomatic treatment; a cross sectional study

Richard Idro[1, 2], Hanifa Namusoke H[1], Catherine Abbo C[3,4], Brian B Mutamba[3,5], Angelina Kakooza-Mwesige[1,6], Robert O Opoka RO[1], Abdu K Musubire[7], Amos D Mwaka AD[7], Bernard T Opar[8]

[1]Department of Paediatrics and Child Health, Mulago hospital/Makerere University College of Health Sciences, Kampala, Uganda

[2]Centre for Tropical Medicine and Global Health, Nuffield Department of Medicine, University of Oxford, UK

[3]Department of Psychiatry, Mulago hospital/Makerere University College of Health Sciences, Kampala, Uganda

[4]Red Cross War Memorial Children's Hospital, Division of Child and Adolescent Psychiatry, University of Cape Town, South Africa.

[5]Butabika National Referral hospital, Kampala, Uganda

[6]Astrid Lindgren Children's Hospital, Neuropediatric Research Unit, Karolinska Institutet, Sweden

[7]Department of Internal Medicine, Mulago hospital/Makerere University College of Health Sciences, Kampala, Uganda

[8]Ministry of Health Headquarters, Kampala, Uganda

Key words

Nodding syndrome, epilepsy, treatment, outcome

Word count

Abstract 298

Full text 3,709

Running title

Treatment Outcomes of Nodding Syndrome in Uganda

Correspondence

Dr Richard Idro

Department of Paediatrics and Child Health, Mulago hospital/Makerere University College of Health Sciences, P.O Box 7072, Kampala, Uganda

Tel: +256 414 531875

Email: ridro1@gmail.com

ABSTRACT

For peer review only - http://bmjopen.bmj.com/site/about/guidelines.xhtml

Treatment Outcomes of Nodding Syndrome in Uganda

Objectives

Nodding syndrome (NS) is a poorly understood neurologic disorder affecting thousands of children in Africa. In March 2012, we introduced a treatment intervention that aimed to provide symptomatic relief. This intervention included sodium valproate for seizures, management of behaviour and emotional difficulties, nutritional therapy and physical rehabilitation. We assessed the clinical and functional outcomes of this intervention after 12 months of implementation.

Design

This was a cross-sectional study of a cohort of patients with NS receiving the specified intervention. We abstracted pre-intervention features from records and compared these to current clinical status. We performed similar assessments on a cohort of patients with other convulsive epilepsies (OCE) and compared outcomes of the two groups.

Participants

Participants were patients with WHO defined NS and patients with OCE attending the same centres.

Outcome measures

The primary outcome was the proportion of patients with seizure freedom (≥1 month without seizures). Secondary outcome measures included reduction in seizure frequency, resolution of behaviour and emotional difficulties, and independence in basic self-care.

Results

Patients with NS had had a longer duration of symptoms (median 5[IQR 3, 6] years) compared to OCE (4[IQR 2, 6] years), $p<0.001$. The intervention resulted in marked improvements in both groups; compared to the pre-intervention state, 121/484(25.0%) patients with NS achieved seizure freedom and there was >70% reduction in seizure frequency; behaviour and emotional difficulties resolved in 194/327(59%); 193/484(40%) had enrolled in school including 17.7% who had earlier withdrawn due to severe seizures and over 80% had achieved independence in basic self care. These improvements were however less than that in patients with OCE of who, 243/476(51.1%) were seizure free and the seizure frequency had reduced by 86%.

Conclusions

Ugandan children with NS show substantial clinical and functional improvements with symptomatic treatments suggesting that NS is probably a reversible encephalopathy.

ARTICLE SUMMARY

Article focus

For peer review only - http://bmjopen.bmj.com/site/about/guidelines.xhtml

Treatment Outcomes of Nodding Syndrome in Uganda

- This paper examines the clinical and functional outcomes of a symptomatic treatment intervention for children with nodding syndrome in Uganda, and compares the improvements to that of patients with other convulsive epilepsies in the same setting.

Key messages

- The symptoms and psychomotor functioning of patients with nodding syndrome improve with symptomatic treatments suggesting that nodding syndrome is probably a reversible epileptic encephalopathy. Symptom reversibility may depend on timing of interventions.
- The improvements are however less than that observed in patients with other convulsive epilepsies suggesting that seizures in nodding syndrome may be less anticonvulsant sensitive compared to seizures in the other convulsive epilepsies.

Strengths and limitations of the study

- This is the largest cohort of patients with nodding syndrome ever reported on to date. The report examines pre-intervention clinical and functional features before a well designed treatment intervention was implemented and how these improved over the course of the ensuing year. The improvements in patients with nodding syndrome were also compared to that of a similar cohort with other epilepsies.
-
- However, we did not conduct a prospective study but rather before and after cross sectional studies meaning that we cannot comment on the incidence of death or loss to follow. We also relied on patient records for the pre-intervention features. Other than head nodding, seizures in nodding syndrome are similar to seizures in other convulsive epilepsies and over time, head nodding may cease in some patients with nodding syndrome increasing the risk of misclassification. In addition, we did not determine compliance to antiepileptic drugs or have reports of adverse effects patients experienced while on treatment and did not have a detailed documentation of the nutritional and cognitive stimulatory treatments each child received. We however limited the effects of such bias by choosing only a few outcome measures that are not easily confused.

BACKGROUND

Nodding syndrome is a poorly understood devastating neurologic disorder affecting several thousand children in the sub-Saharan African countries of South Sudan[1-3], Uganda[4-6] and

For peer review only - http://bmjopen.bmj.com/site/about/guidelines.xhtml

Treatment Outcomes of Nodding Syndrome in Uganda

Tanzania.[7-9] The syndrome is characterised by almost daily atonic seizures manifesting as clusters of head nods[4] and complicated by tonic clonic, focal, myoclonic and/or atypical absence seizures, cognitive and motor decline, malnutrition, behavioural and emotional difficulties[6, 7]. The aetiology is unknown although the syndrome has been associated with infestation with *Onchocerca volvulus.*[1, 5, 7] Studies of Tanzanian and Ugandan patients have concluded that nodding syndrome is probably symptomatic generalised epilepsy.[4, 6, 7]

In Uganda, a multidisciplinary team developed management guidelines for care[10]. The objective was to relieve symptoms, offer primary and secondary prevention of disability, and rehabilitation to improve function. The most important clinical needs were identified as seizure control, relief of behavioural and emotional difficulties, nutritional therapy, physical and cognitive rehabilitation. The first group of patients were enrolled in March 2012. We evaluated clinical outcomes of this intervention after a minimum of 12 months. We hypothesised that if treated with appropriate anticonvulsants, patients with nodding syndrome would achieve similar seizure control like patients with other convulsive epilepsies. We therefore in addition, compared outcomes of patients with nodding syndrome to that in patients with other convulsive epilepsies.

For peer review only - http://bmjopen.bmj.com/site/about/guidelines.xhtml

Treatment Outcomes of Nodding Syndrome in Uganda

METHODS

Design and setting

This was a cross sectional survey of a cohort of patients with nodding syndrome that evaluated the clinical and functional outcomes of patients receiving the Ugandan Ministry of Health treatment intervention at least 12 months after initiation of therapy. We performed a similar evaluation on a cohort of patients with other convulsive epilepsies that attended the same centres and compared improvements in the two groups. The study was conducted in northern Uganda, the region most affected by nodding syndrome in the country. The population prevalence of probable nodding syndrome among children of the affected age group in the study area has been estimated as 6.8 (95% CI 5.9 – 7.7) per 1,000[11]. This region also suffered a protracted armed rebellion that lasted over 20 years[12] resulting into massive internal displacement. It is only in the past 6-7 years that peace prevailed and the population returned to their homes.

Participants

Participants were patients with either nodding syndrome or other convulsive epilepsies receiving treatment at any one of the nodding syndrome treatment centres in the seven districts of Lamwo, Kitgum, Pader, Gulu, Amuru, Lira and Oyam. The definition of head nodding and diagnosis of nodding syndrome is in accord with the criteria developed by international consensus during the World Health Organization facilitated meeting on Nodding Syndrome in Kampala, 2012[13]. Head nodding was defined as repetitive, involuntary drops of the head on to the chest in previously normal persons. We included probable and confirmed cases only. Children with other convulsive epilepsies were those with active (at least one in the past year) tonic-clonic or focal jerking epileptic seizures. The diagnosis and classification of epilepsy in this rural community is quite limited and in many cases, categorisation into specific clinical groups is not possible. We therefore only included those with convulsive epilepsies. Participants with onset of symptoms outside of the ages 3-18 years were excluded to allow comparability with nodding syndrome patients.

The intervention

The nodding syndrome treatment centres in Lamwo, Kitgum and Pader were opened in March 2012 followed by those in Amuru, Gulu, Lira and Oyam in June 2012. Prior to this, clinicians and nurses at each centre underwent a five-day training on the management of nodding syndrome using the specified guideline[14]. The training which also included general principles of epilepsy treatment was provided through didactic lectures, role play, bedside clinical teachings and demonstrations by the same team that developed the guidelines. At the end of the five days, each

For peer review only - http://bmjopen.bmj.com/site/about/guidelines.xhtml

team returned to their centre and worked with the trainers to initiate provision of care. Other than the centre in Kitgum which is a district hospital (a level V health centre), all the others were health centre III. At each centre, clinical service was led by a medical or psychiatric clinical officer (individuals with a diploma in clinical medicine or psychiatry after three years of training), general and psychiatric nurses, laboratory technicians and either a physiotherapist or occupational therapist. In Kitgum hospital, the team was led by a medical officer (MBChB). These teams were supported by local lay volunteers - village health workers – who coordinated follow up and ambulatory care in homes. In each district, supervisory oversight was provided by a district nodding syndrome focal person, the District Health Officer and the district nodding syndrome committee while nationally, there was a national nodding syndrome coordinator who brought everyone together. Over the next 12 months, each centre received support supervision visits on at least two occasions to maintain skills and attend to issues arising.

Details of the treatment are described elsewhere[10]. In summary, inpatient emergency care was offered to patients with life threatening co-morbidities. Ambulatory and community care was offered to patients without co-morbidities or those with non-life threatening co-morbidities. Sodium valproate was the first-line anticonvulsant starting at 10mg/kg/day in two divided doses and the dose titrated to a maximum of 40mg/kg/day. The patient's family was provided with supplemental food rations every 2-4 weeks. Severely malnourished patients with medical complications were treated as inpatients and those with uncomplicated severe malnutrition were treated as outpatients with ready to use therapeutic feeds. This was provided as Plumpy'Nut® a product of Nutriset, (Normandy, France). Plumpy'Nut is made of peanut paste, vegetable oil, powdered milk and sugar, vitamins (A, B-complex, C, D, E and K) and minerals (calcium, phosphorus, potassium, magnesium, zinc, copper, iron, iodine, sodium and selenium) all combined in a foil pouch. Each 92g pack provides 500 calories. Management of behaviour and emotional difficulties included counselling and referral of those with severe symptoms to mental health services. Other management included physical, speech and language therapy and cognitive stimulation.

Children with other convulsive epilepsies were provided with first-line anticonvulsants (carbamazepine, phenobarbitone, phenytoin or sodium valproate) or continued to receive earlier prescribed anticonvulsants but the dose adjusted appropriately. A new anticonvulsant was introduced if an inappropriate drug was being provided. Anticonvulsants such as oxcarbazepine, lamotrigine, levetiracetam, and topiramate are unavailable in the public health service in Uganda. Families of patients with other convulsive epilepsies were also provided with similar supplemental

For peer review only - http://bmjopen.bmj.com/site/about/guidelines.xhtml

feeding. In addition, parents/carers of both groups of patients were educated on seizures, epilepsy, adherence to antiepileptic drugs and prevention of seizure related injuries.

Sample size

In a preliminary evaluation of the treatment outcomes of nodding syndrome after seven months of intervention, we documented (from parental or carer report) that 5/47 (10%) had achieved seizure freedom (no head nodding or convulsive seizures) for at least 30 days prior to the visit. Using these findings, we estimated that a sample of 432 patients will be sufficient at 5% level of significance and 90% power to detect a 10% increase in this proportion to 20% after 12 months of treatment. Secondly, up to 70% of children with new onset convulsive epilepsies achieve terminal seizure remission with drug treatment.[15, 16] The onset of seizure remission is often evident within the first year of treatment[15]. Using these findings, we estimated that with a sample of 461 nodding syndrome patients and a similar number with other convulsive epilepsies, we will be able to, at 90% power and 5% level of significance, reject the null hypothesis that there is no difference in the proportions of patients with nodding syndrome or other convulsive epilepsies achieving seizure freedom with 12 months therapy. We set to recruit the larger sample.

Study procedures and measurements

As of 30 June 2013, there were 3,295 patients with probable or confirmed nodding syndrome receiving care at the seven centres. We used proportionate sampling to estimate the number of participants to be recruited from each centre and consecutively recruited patients as they presented until the sample was achieved. Data was collected between 1st July 2013 and 30th September 2013. One of two investigators (RI or BTO) first conducted a day's training on the study procedures followed by a joint clinic with the clinicians at the centre. The local clinical team subsequently worked independently until study completion. Case record forms were completed from data abstracted from pre-intervention records, direct inquiry from parents/carers and on physical exam. The pre-intervention seizure burden, weight and height, and behaviour or emotional difficulty was obtained from records. We defined seizures as head nodding or convulsive seizures and defined seizure burden as the number of clusters of head nodding and/or convulsive seizures per unit time.

In the clinic, parents/carers reported on current seizures, behaviour and emotional difficulties. Weight was measured using a stand on electronic scale while height or length was measured

For peer review only - http://bmjopen.bmj.com/site/about/guidelines.xhtml

using a stadiometer. Independence in basic self care (self feeding, dressing and using a toilet), the status of schooling, and ability to appropriately help with culturally and age-appropriate homecare activities (e.g. sweeping the compound) were obtained from the parents or carers. The parents and carers were also asked to provide an overall assessment of improvements or worsening of symptoms over the year on an ordinal scale (markedly improved, some improvement, no improvement or worse).

Outcome measures

The primary outcome was the proportion of patients who had achieved seizure freedom (defined as ≥1 month without seizures [no head nodding and/or convulsive seizures observed by the parent/carer prior to follow up visit]). Secondary outcomes included reduction in seizure burden (reduction in the mean number of clusters of head nods and/or convulsive seizures per unit time), the proportions of patients with independence in basic self care, resolution of behaviour and emotional difficulties, and enrolment in school.

Data management and statistical analysis

Data was collected on case record forms and double-entered into a Microsoft Access 2007 database. Data analysis was performed using STATA version 12.0 (STATA Corp, Tx). The two patient groups were considered as two independent single samples and paired data (before initiation of therapy and at least 12 months later) analysis was performed for each group. Thus, we determined the proportions of patients with nodding syndrome with seizure freedom before and after 12 months and the proportions with the different secondary outcomes. A one sample t-test was used to compare means of normally distributed continuous data, the Mann-Whitney U test for medians of skewed data and McNemar's test for categorical data. We then examined for patient characteristics potentially associated with seizure freedom including duration and age at onset of symptoms, baseline seizure frequency, presence of behaviour and emotional difficulties, whether the child had head nodding only or head nodding plus (other seizures) and antiepileptic drug dose and performed a logistic regression analysis to determine variables independently associated with achieving seizure freedom.

RESULTS

General descriptions

A total of 1,322 subjects were screened in six out of the seven districts. Oyam district, which had only eight patients with nodding syndrome, was not visited. Two hundred and fifteen subjects

For peer review only - http://bmjopen.bmj.com/site/about/guidelines.xhtml

For peer review only

were ineligible. Another 147 were also excluded for different reasons. Thus, 960 participants (484 with nodding syndrome and 476 with other convulsive epilepsies) were available for the study (**figure 1**).

The two groups were of similar age and gender; the mean (SD) age of patients with nodding syndrome was 13.7(3.6) years and that for patients with other convulsive epilepsies was 13.0(2.9) years, p=0.998; 281/484 (58.1%) subjects with nodding syndrome and 267/476 (56.1%) with other convulsive epilepsies were male, p=0.538. However, participants with nodding syndrome had experienced a longer duration of symptoms (median 5[IQR 3, 6] years) compared to patients with other epilepsies, (median 4 [IQR 2, 6] years), p<0.001.

The median daily dose of sodium valproate in patients with nodding syndrome was 16(IQR 12, 21) mg/kg/day with most (298/484, 61.6%) on relatively low doses (<20mg/kg/day). The majority of the patients with other convulsive epilepsies (421/476, 88.5%) were on carbamazepine, phenobarbitone or phenytoin monotherapy. The remaining 55 were either on sodium valproate (40/476, 8.4%) or combinations of the above anticonvulsants (15/476, 3.1%).

Outcomes of interventions

a) Seizures

There was marked reduction in seizures with the intervention; overall, 25% (95% CI 21, 29) of nodding syndrome patients achieved seizure freedom. Both the frequency of head nodding and of convulsive seizures reduced by over 70%. The reduction in seizure burden was even more marked in patients with other convulsive epilepsies; 51% (95% CI 46.4, 55.6) achieved seizure freedom and the overall burden of seizures decreased by 86%, **(table 1)**.

Although the effects of sodium valproate on seizure control in nodding syndrome was evident at relatively low doses, additional patients achieved seizure freedom with dose escalation. Thus, 87/298 (29.2%) patients were seizure free on sodium valproate <20mg/kg/day and an additional 34/186 (18.3%) achieved seizure freedom with dose increases to 20-40mg/kg/day.

For peer review only - http://bmjopen.bmj.com/site/about/guidelines.xhtml

Treatment Outcomes of Nodding Syndrome in Uganda

Table 1 Pre-interventions features and features at least 12 months after initiation of a symptomatic treatment intervention in patients with nodding syndrome or other convulsive epilepsies

	Patients with nodding syndrome, N=484			Other convulsive epilepsies, N=476		
	**Pre- intervention status**	**Features ≥ 12 months later**	**P value**	**Pre-intervention status**	**Features ≥ 12 months later**	**P value**
**Patients with seizure freedom*, %**	8 (2%) [95% CI 0.07, 3.2]	121 (25.0%) [95% CI 21.2, 29.1]	<0.001	8 (2%) [95% CI 0.7, 3.3]	243 (51.1%) [95% CI 46.4, 55.6]	<0.001
**Daily clusters of head nods, median (IQR)**	4 (IQR 3, 6)	1 (IQR 0, 2)	<0.001	-	-	-
**Patients with behaviour & emotional difficulties, %**	327/484 (67.6%) [95% CI 63.2, 71.7]	133 (27.5%) [95% CI 23.5, 33.7]	<0.001	250/476 (52.5%) [95% CI 47.9, 57.1]	105 (22.1%) [95% CI 18.4, 26.1]	<0.001
**GMFCS score****						
**1**	185/282 (64.0%)	223/282 (79.1%)	<0.001***	212/288 (73.6%)	239/288 (83.0%)	<0.001***
**2**	58/282 (20.1%)	39/282 (13.8%)		41/288 (14.1%)	39/288 (13.5%)	
**3**	39/288 (13.5%)	39/288 (13.5%)		39/288 (13.5%)	10/288 (3.5%)	
**4 and 5**	39/288 (13.5%)	0 (0)		2/288 (0.7%)	0 (0)	
**Independence in basic self care, %**	174 (36.0%) [95% CI 31.7, 40.4]	402 (83.1%) [95% CI 79.4, 86.3]	< 0.001	206 (43.3%) [95% CI 38.8, 47.9]	397 (83.4%) [95% CI 79.8, 86.6]	<0.001
**Able and performs culturally and age appropriate homecare activities, %**	152 (31.4%) [95% CI 27.2, 37.4]	372 (76.9%) [95% CI 72.8, 80.5]	<0.001	187 (39.3%) [95% CI 34.9, 43.8]	382 (80.3%) [95% CI 76.4, 83.7]	<0.001
**Enrolled at and attending school, %**	107 (22.1%) [95% CI 18.5, 26.1]	193 (39.9%) [95% CI 35.5, 44.4]	<0.001	170 (35.7%) [95% CI 31.4, 40.2]	250 (52.5%) [95% CI 47.9, 57.1]	<0.001

*≥1 month without seizures
**GMFCS=Gross Motor Function Classification Score; N=282; i.e. Only 282 patients with nodding syndrome had paired GMFCS pre and post interventions scores obtained.
***Chi square test for trend with Yate's correction

We repeated diagnostic electroencephalogram (EEG) recordings for three patients with nodding syndrome who were part of the 22 we reported on earlier[6]. The recordings showed clear improvements in background EEG and reductions in previously widespread interictal epileptiform discharges. All three were on sodium valproate 20-25mg/kg/day and were experiencing only occasional convulsive seizures but no head nodding.

For peer review only - http://bmjopen.bmj.com/site/about/guidelines.xhtml

Treatment Outcomes of Nodding Syndrome in Uganda

b) Behaviour and emotional difficulties

Behaviour and emotional difficulties were reported in 327(67.6%) participants with nodding syndrome and in 250(52.5%) with other convulsive epilepsies prior to the intervention. Among participants with nodding syndrome, these included aggressive and destructive behaviour (186/484, 39.5%), wandering or running away (113/484, 23.4%) and periods of low mood (114/484, 23.6%). Over the 12 months, the difficulties resolved in 194/327 (59.3%) nodding syndrome patients and in 145/250 (58.0%) patients with other convulsive epilepsies. Improvements were most evident in nodding syndrome patients initially reporting wandering, aggressive and destructive behaviour. Psychotropic drugs (haloperidol) were prescribed for only three patients with severe difficulties and two received anxiolytic drugs. An additional 62(12.8%) nodding syndrome patients, especially those with uncontrolled or worsening seizures, developed new onset behaviour and emotional difficulties; these included 44 (9.1%) aggressive and destructive behaviour, 18(3.7%) wandering, and 21(4.3%) mood problems. Wandering behaviour was uncommon among patients with other convulsive epilepsies in whom impulsive behaviour and hyperactivity were more common.

c) Independence in basic self care

Prior to the intervention, 174/484(36.0%) patients with nodding syndrome were independent in basic self care. This proportion had increased to 402/484(83.1%) by the time of the survey, $p<0.001$. Similar improvements were observed in patients with other convulsive epilepsies. Thus, 397/476(83.4%) of these patients were independent in basic self care at the time of the survey up from 270/476 (56.7%) prior to intervention, $p<0.001$.

d) School of attendance

A total of 443 patients (193/484, 39.9% with nodding syndrome and 250/476, 52.5% with other convulsive epilepsies) were enrolled in and attending school at the time of the survey. This included 86/484 (17.8%) patients with nodding syndrome and 80/476 (16.8%) patients with other convulsive epilepsies who had returned to school with seizure control and improvements in other symptoms. Although these children had returned to school, parents reported that 90/193(46.6%) patients with nodding and 76/250(30.4%) patients with other epilepsies were still performing poorly in school.

For peer review only - http://bmjopen.bmj.com/site/about/guidelines.xhtml

Treatment Outcomes of Nodding Syndrome in Uganda

e) Qualitative assessment of improvements by parents and carers

On an ordinal subjective scale, parents felt that 112/484(23.1%) patients with nodding syndrome and 253/476(53.2%) patients with other convulsive epilepsies had improved markedly. Another 325/484(67.2%) patients with nodding syndrome and 194/476(40.8%) with other convulsive epilepsies had some improvement. The number of patients with nodding syndrome who could participate and help their parents with home care tasks increased from 152/484 (31.4%) to 372/484 (76.9%) with the intervention. Only 47/484(9.7%) patients with nodding syndrome and 29/476(6.1%) with other convulsive epilepsies had no improvement in symptoms or became worse over the period of intervention.

Prognostic factors for seizure freedom

While we examined the relationship between gender, age at onset of symptoms, duration of symptoms, baseline seizure frequency, presence of behaviour and emotional difficulties, whether the child had head nodding only or head nodding plus (other seizures), antiepileptic drug dose and achieving seizure freedom, only a lower number of clusters of head nods prior to the intervention (adjusted OR 0.80 [95% CI 0.72-0.88], $p<0.001$) and response to a lower antiepileptic drug dose (adjusted OR 0.96 (95% CI 0.93, 0.99], p=0.046) were independently associated with achieving seizure freedom.

DISCUSSION

Our study aimed to determine the clinical outcomes and therefore, the effectiveness of a symptomatic treatment intervention for nodding syndrome. We documented substantial clinical and functional improvements with the intervention. The findings suggest that nodding syndrome is probably a reversible encephalopathy. The improvements we observed were however less than that seen in patients with other convulsive epilepsies suggesting that epileptic seizures in nodding syndrome may be less anticonvulsant sensitive compared to seizures in the other convulsive epilepsies.

Although the proportion of patients with nodding syndrome who achieved seizure freedom was modest, our findings suggest that a treatment package of selected anticonvulsants, psycho-behavioural interventions and nutritional and physical rehabilitation can control seizures, improve function and even reverse some severe functional disability in nodding syndrome. This observation seems to concur with a report from Tanzania in which although seizure freedom was achieved by 2/32 patients treated with phenobarbitone, over 80% had reductions in seizure

For peer review only - http://bmjopen.bmj.com/site/about/guidelines.xhtml

burden..[9] Even though we did not perform specific cognitive testing or brain imaging to objectively document functional and structural improvements with the intervention, comparisons of a pre-intervention and repeat EEG recordings in three patients with previous recordings demonstrated clear improvements in background EEG and reductions in the previously widespread interictal epileptiform discharges.[6]

Clinical trials comparing treatment of seizures in nodding syndrome with sodium valproate to treatment with other anticonvulsants such as lamotrigine or levetiracetam either as monotherapy or as add on therapy may be considered. In addition and especially for patients whose symptoms are either not controlled or became worse on therapy, other strategies may be considered. Epileptic encephalopathy is a possibility especially in patients with severe and persistent symptoms. Can therapy with benzodiazepines, high dose steroids or other immunosuppressant drugs be considered?[17]

The aetiology of nodding syndrome is still unknown. In all three countries where nodding syndrome has been described, it has been associated with infestation by *Onchocerca volvulus*.[1, 7, 18] Uganda is in its second year of twice yearly mass administration of ivermectin (an antimicrofilarial agent active only against the mirofilaria but not the adult parasite). Other strategies that target the both microfiliaria and the adult worms and/or their co-symbiotic bacteria, Wolbachiae, may be considered as potential specific therapy.[19, 20]

Despite these improvements, parents reported that the majority of the 40% children who returned to school continued to perform poorly. There is need to examine whether the continued poor academic performance is due to irreparable brain injury or an underlying ongoing aetio-pathogenic process. To date, there are no systematic studies of cognitive function in nodding syndrome. Such studies will help define areas of functional deficits and document improvements preferably using tools that can be applied across different regions with minimal modification to allow comparison.

We did not apply specific psychiatric diagnostic tools to patients with behaviour and emotional difficulties to be able to make distinct psychiatric diagnoses. A few children with severe difficulties were attended to by the local mental health services and some given psychotropic drugs. The majority of the 194 children in whom behaviour and emotional difficulties resolved however improved without psychotropic drugs but with seizure control suggesting that in nodding

For peer review only - http://bmjopen.bmj.com/site/about/guidelines.xhtml

syndrome, some of these features may be co-morbidities of epilepsy. Wandering behaviour may be an ictal event[6]. Some patients may also have benefitted from the effects of sodium valproate on behaviour; in a recent case series of Ugandan children, Musisi et al documented improvements in some patients receiving antidepressants.[21] Put together, these findings suggest that psychotropic drugs may be considered for some patients with nodding syndrome especially those with severe symptoms.

Study limitations

First, other than head nodding, seizures in nodding syndrome may be similar to seizures in other convulsive epilepsies and over time, head nodding may cease in some patients.[9] This scenario opens room for potential misclassification of disease as the current disease criteria heavily leans on clinical observations. Secondly, we did not perform a prospective study; instead, we relied on patient records for pre-intervention features. Third, we had only limited data on the burden and severity of other co-morbidities such as injuries (e.g. burns) or earlier exposure to acute encephalopathies such as cerebral malaria, meningitis and encephalitis. Fourth, participants had varied periods of exposure to the intervention, a factor that may have affected the estimate of the effect. Fifth, we did not determine compliance to antiepileptic drugs or have reports of adverse effects patients experienced while on treatment. We also did not have a detailed documentation of the nutritional therapy and the cognitive stimulatory activities each child received and did not assess the effect of home environment on outcome. We however limited the effects of such bias by choosing only few and fairly robust outcome measures.

Failure to conduct a prospective study means that we cannot comment on the incidence of death or on patients who might have discontinued follow up care (e.g. due to deterioration in symptoms, severe motor disability or loss of faith in the treatment) leading to an over estimate of the effect. Such an effect if any is likely minimal. From Ministry of Health epidemiological surveillance reports, only 12 patients with probable nodding syndrome died over the period of observation mostly from seizure related events.

Furthermore, our comparative group – participants with other convulsive epilepsies – were a heterogeneous group with different seizure types and possibly neuropathology, on treatment with different anticonvulsants each with different efficacy, dose and side effects. It would have served us better to recruit a more homogenous group of patients, for example, only patients with generalised seizures on treatment with a single anticonvulsant. However, in this rural community,

For peer review only - http://bmjopen.bmj.com/site/about/guidelines.xhtml

the diagnosis of epilepsy is only limited to clinical features obtained on history and clinical observations by clinicians with limited training. Despite this weakness, our results clearly demonstrate that the outcome of nodding syndrome is different from that of the combined heterogeneous group of patients with the other convulsive epilepsies.

Conclusions

The symptoms and psychomotor functioning of patients with nodding syndrome improve with symptomatic treatments suggesting that nodding syndrome is probably a reversible epileptic encephalopathy. Symptom reversibility may depend on the timing of interventions. Uncontrolled epileptic seizures may be a major contributor to the neuro-cognitive decline and disability in this syndrome. Further studies to elucidate these findings recommended.

For peer review only

For peer review only - http://bmjopen.bmj.com/site/about/guidelines.xhtml

Treatment Outcomes of Nodding Syndrome in Uganda

Author's contributions

RI, HN, CA, BBM, AKM, ROO, AKM, ADM and BTO designed the intervention and the study and participated in supervising patient care. RI and HN performed the data analysis. RI wrote the first draft and all participated in data interpretation and provided a critical review of the manuscript.

Funding

The study was funded by the Government of Uganda. Dr Idro is partly supported by the Wellcome Trust through a Director's discretionary research funds. The funding agency had no role in data collection, analysis and interpretation of results and the decision to submit for publication.

Competing interests

There are no conflicts of interest to report.

Ethics approval

Makerere University School of Medicine Research and Ethics Committee

Data sharing statement

No additional data are available.

Acknowledgements

We would like the thank Local District Health Services in Lamwo, Kitgum, Pader, Amuru, Gulu, and Lira who made the study possible, Keron Ssebyala who managed the data and Prof Gretchen Birbeck, who critically reviewed the first draft of the this manuscript and offered helpful comments to improve it.

For peer review only

Treatment Outcomes of Nodding Syndrome in Uganda

Reference

1. Tumwine JK, Vandemaele K, Chungong S, Richer M, Anker M, Ayana Y, et al. Clinical and epidemiologic characteristics of nodding syndrome in Mundri County, southern Sudan. African health sciences. 2012; 12(3): 242-8.
2. Spencer PS, Vandemaele K, Richer M, Palmer VS, Chungong S, Anker M, et al. Nodding syndrome in Mundri county, South Sudan: environmental, nutritional and infectious factors. African health sciences. 2013; 13(2): 183-204.
3. Nyunguraa JL, Akimb, T., Lakoc, A., Gordond, A., Lejenge, L., William, G. Investigation into the Nodding syndrome in Witto Payam, Western Equatoria State, 2010. Southern Sudan Medical Journal. 2011 4(1): 3-6.
4. Sejvar JJ, Kakooza AM, Foltz JL, Makumbi I, Atai-Omoruto AD, Malimbo M, et al. Clinical, neurological, and electrophysiological features of nodding syndrome in Kitgum, Uganda: an observational case series. Lancet neurology. 2013; 12(2): 166-74.
5. Foltz JL, Makumbi I, Sejvar JJ, Malimbo M, Ndyomugyenyi R, Atai-Omoruto AD, et al. An Epidemiologic Investigation of Potential Risk Factors for Nodding Syndrome in Kitgum District, Uganda. PloS one. 2013; 8(6): e66419.
6. Idro R, Opoka RO, Aanyu HT, Kakooza-Mwesige A, Piloya-Were T, Namusoke H, et al. Nodding syndrome in Ugandan children--clinical features, brain imaging and complications: a case series. BMJ open. 2013; 3(5).
7. Winkler AS, Friedrich K, Konig R, Meindl M, Helbok R, Unterberger I, et al. The head nodding syndrome--clinical classification and possible causes. Epilepsia. 2008; 49(12): 2008-15.
8. Winkler AS, Friedrich K, Meindl M, Kidunda A, Nassri A, Jilek-Aall L, et al. Clinical characteristics of people with head nodding in southern Tanzania. Tropical doctor. 2010; 40(3): 173-5.
9. Winkler AS, Wallner B, Friedrich K, Pfausler B, Unterberger I, Matuja W, et al. A longitudinal study on nodding syndrome--a new African epilepsy disorder. Epilepsia. 2014; 55(1): 86-93.
10. Idro R, Musubire KA, Byamah Mutamba B, Namusoke H, Muron J, Abbo C, et al. Proposed guidelines for the management of nodding syndrome. Afr Health Sci. 2013; 13(2): 219-32.
11. Iyengar PJ, Wamala J, Ratto J, Blanton C, Malimbo M, Lukwago L, et al. Prevalence of nodding syndrome--Uganda, 2012-2013. MMWR Morbidity and mortality weekly report. 2014; 63(28): 603-6.
12. Wendo C. Northern Uganda humanitarian crisis shocks UN chief. Rebels in northern districts have left people trapped in hunger, disease, poverty, and fear. Lancet. 2003; 362(9398): 1818.
13. WHO. International Scientific Meeting on Nodding Syndrome. Geneva: World Health Organization; 2012.
14. Idro R, Musubire KA, Byamah Mutamba B, Namusoke H, Muron J, Abbo C, et al. Proposed guidelines for the management of nodding syndrome. African health sciences. 2013; 13(2): 219-32.
15. Hauser E, Freilinger M, Seidl R, Groh C. Prognosis of childhood epilepsy in newly referred patients. Journal of child neurology. 1996; 11(3): 201-4.
16. Berg AT, Shinnar S, Levy SR, Testa FM, Smith-Rapaport S, Beckerman B, et al. Two-year remission and subsequent relapse in children with newly diagnosed epilepsy. Epilepsia. 2001; 42(12): 1553-62.
17. Parisi P, Spalice A, Nicita F, Papetti L, Ursitti F, Verrotti A, et al. "Epileptic encephalopathy" of infancy and childhood: electro-clinical pictures and recent understandings. Current neuropharmacology. 2010; 8(4): 409-21.
18. Nodding syndrome - South Sudan, 2011. MMWR Morb Mortal Wkly Rep. 2012; 61(3): 52-4.
19. Hoerauf A, Specht S, Buttner M, Pfarr K, Mand S, Fimmers R, et al. Wolbachia endobacteria depletion by doxycycline as antifilarial therapy has macrofilaricidal activity in onchocerciasis: a randomized placebo-controlled study. Medical microbiology and immunology. 2008; 197(3): 295-311.

For peer review only - http://bmjopen.bmj.com/site/about/guidelines.xhtml

Treatment Outcomes of Nodding Syndrome in Uganda

20. Hoerauf A, Mand S, Adjei O, Fleischer B, Buttner DW. Depletion of wolbachia endobacteria in Onchocerca volvulus by doxycycline and microfilaridermia after ivermectin treatment. Lancet. 2001; 357(9266): 1415-6.

21. Musisi S, Akena D, Nakimuli-Mpungu E, Abbo C, Okello J. Neuropsychiatric perspectives on nodding syndrome in northern Uganda: a case series study and a review of the literature. African health sciences. 2013; 13(2): 205-18.

For peer review only

For peer review only - http://bmjopen.bmj.com/site/about/guidelines.xhtml

Treatment Outcomes of Nodding Syndrome in Uganda

Nodding syndrome patients in Uganda improve with symptomatic treatment; a cross sectional study

Richard Idro[1, 2], Hanifa Namusoke H[1], Catherine Abbo C[3,4], Brian B Mutamba[3,5], Angelina Kakooza-Mwesige[1,6], Robert O Opoka RO[1], Abdu K Musubire[7], Amos D Mwaka AD[7], Bernard T Opar[8]

[1]Department of Paediatrics and Child Health, Mulago hospital/Makerere University College of Health Sciences, Kampala, Uganda

[2]Centre for Tropical Medicine and Global Health, Nuffield Department of Medicine, University of Oxford, UK

[3]Department of Psychiatry, Mulago hospital/Makerere University College of Health Sciences, Kampala, Uganda

[4]Red Cross War Memorial Children's Hospital, Division of Child and Adolescent Psychiatry, University of Cape Town, South Africa.

[5]Butabika National Referral hospital, Kampala, Uganda

[6]Astrid Lindgren Children's Hospital, Neuropediatric Research Unit, Karolinska Institutet, Sweden

[7]Department of Internal Medicine, Mulago hospital/Makerere University College of Health Sciences, Kampala, Uganda

[8]Ministry of Health Headquarters, Kampala, Uganda

Key words

Nodding syndrome, epilepsy, treatment, outcome

Word count

Abstract	~~299~~298
Full text	3,~~449~~709

Running title

Treatment Outcomes of Nodding Syndrome in Uganda

Correspondence

Dr Richard Idro

Department of Paediatrics and Child Health, Mulago hospital/Makerere University College of Health Sciences, P.O Box 7072, Kampala, Uganda

Tel: +256 414 531875

Email: ridro1@gmail.com

For peer review only

Treatment Outcomes of Nodding Syndrome in Uganda

ABSTRACT

Objectives

Nodding syndrome (NS) is a poorly understood neurologic disorder affecting thousands of children in Africa. In March 2012, w~~W~~e introduced a ~~symptomatic~~ treatment intervention ~~in Uganda~~that aimed to provide symptomatic relief~~ in March 2012~~. This intervention included sodium valproate for seizures, management of behaviour and emotional difficulties, nutritional therapy and physical rehabilitation. We assessed the clinical and functional outcomes of ~~the~~ this intervention after ~~at least~~ 12 months of implementation.

Design

This was a cross-sectional study of a cohort of patients with NS receiving the specified intervention. We abstracted pre-intervention features from records and compared these to current clinical status. We performed similar assessments on a cohort of patients with other convulsive epilepsies (OCE) and compared outcomes of the two groups.

Participants

Participants were patients with WHO defined NS and patients with OCE attending the same ~~treatment~~ centres.

Outcome measures

The primary outcome was the proportion of patients with seizure freedom (≥1 month without seizures). Secondary outcome measures included reduction in seizure frequency, resolution of behaviour and emotional difficulties, and independence in basic self-care.

Results

Patients with NS had had a longer duration of symptoms (median 5[IQR 3, 6] years) compared to OCE (4[IQR 2, 6] years), *p*<0.001. ~~Among 484 patients with NS and 476 with OCE, t~~The intervention resulted in marked improvements in both groups; compared to the pre-intervention state, 121/484(25.0%) patients with NS achieved seizure freedom and there was >70% reduction in seizure frequency; behaviour and emotional difficulties resolved in 194/327(59%); 193/484(40%) had enrolled in school including 17.7% who had earlier withdrawn due to severe seizures and over 80% had achieved independence in basic self care. These improvements were however less than that in patients with OCE of who, 243/476(51.1%) were seizure free and the seizure frequency had reduced by 86%.

Conclusions

Ugandan children with NS show substantial clinical and functional improvements with symptomatic treatments suggesting that NS is probably a reversible encephalopathy~~treatable~~

Formatted: Font: (Default) Arial

Field Code Changed

For peer review only - http://bmjopen.bmj.com/site/about/guidelines.xhtml

Treatment Outcomes of Nodding Syndrome in Uganda

disorder. Uncontrolled seizures may be a major contributor to the neuro-cognitive decline in this syndrome.

ARTICLE SUMMARY

Article focus

- This paper examines the clinical and functional outcomes of a symptomatic treatment intervention for children withof nodding syndrome in Uganda, and compares the improvementsse outcomes to that of patients with other convulsive epilepsies in the same setting.

Key messages

- The symptoms and psychomotor functioning of patients with nodding syndrome improve with symptomatic treatments suggesting that nodding syndrome is probably a reversible epileptic encephalopathy. Symptom reversibility may depend on timing of interventions.
- The improvements are however less than that observed in patients with other convulsive epilepsies suggesting that seizures in nodding syndrome may be less anticonvulsant sensitive compared to seizures in the other convulsive epilepsies.

Strengths and limitations of the study

- This is the largest cohort of patients with nodding syndrome ever reported on to date. The report examines pre-intervention clinical and functional features before a well designed treatment intervention was implemented and how these improved over the course of the ensuing year. The improvements in patients with nodding syndrome weare also compared to that of a similar cohort with other epilepsies.
-
- However, we did not conduct a prospective study but rather before and after cross sectional studies meaning that we cannot comment on the incidence of death or on loss to follow. We alsoIn addition, we relied on patient records for the pre-intervention features. Other than head nodding, seizures in nodding syndrome are similar to seizures in other convulsive epilepsies and over time, head nodding may cease in some patients with nodding syndrome increasing the risk of misclassification. In addition, we did not determine compliance to antiepileptic drugs or have reports of adverse effects patients experienced while on treatment and did not have a detailed documentation of the nutritional and cognitive stimulatory treatments each child received. We however limited

Formatted: Font: (Default) Arial

For peer review only

Treatment Outcomes of Nodding Syndrome in Uganda

the effects of such bias by choosing only a few outcome measures that are not easily confused.

BACKGROUND

Nodding syndrome is a poorly understood devastating neurologic disorder affecting several thousand children in the sub-Saharan African countries of South Sudan[1-3], Uganda[4-6] and Tanzania.[7-9]~~[3-8]~~ The syndrome is characterised by almost daily atonic seizures manifesting as clusters of head nods[4] and complicated by tonic clonic, focal, myoclonic and/or atypical absence seizures, cognitive and motor decline, malnutrition, behavioural and emotional difficulties[6, 7]. The aetiology is unknown although the syndrome has been associated with infestation with *Onchocerca volvulus.*[1, 5, 7] Studies of Tanzanian and Ugandan patients have concluded that nodding syndrome is probably symptomatic generalised epilepsy.[4, 6, 7]

In Uganda, a multidisciplinary team developed management guidelines for care[10]. The objective was to relieve symptoms, offer primary and secondary prevention of disability, and rehabilitation to improve function. The most important clinical needs were identified as seizure control, relief of behavioural and emotional difficulties, nutritional therapy, physical and cognitive rehabilitation. The first group of patients were enrolled in March 2012. We evaluated clinical outcomes of this intervention after a minimum of 12 months. We hypothesised that if treated with appropriate anticonvulsants, patients with nodding syndrome would achieve similar seizure control like patients with other convulsive epilepsies. We therefore in addition, compared outcomes of patients with nodding syndrome to that in patients with other convulsive epilepsies.

For peer review only

For peer review only - http://bmjopen.bmj.com/site/about/guidelines.xhtml

Treatment Outcomes of Nodding Syndrome in Uganda

METHODS

Design and setting

This was a cross sectional survey of a cohort of patients with nodding syndrome that evaluated the clinical and functional outcomes of patients receiving the Ugandan Ministry of Health treatment intervention at least 12 months after initiation of therapy. We performed a similar evaluation on a cohort of patients with other convulsive epilepsies that attended the same centres and compared improvements in the two groups. The study was conducted in northern Uganda, the region most affected by nodding syndrome in the country. The population prevalence of probable nodding syndrome among children of the affected age group in the study area has been estimated as 6.8 (95% CI 5.9 – 7.7) per 1,000[11]. This region also suffered a protracted armed rebellion that lasted over 20 years[12] resulting into massive internal displacement. It is only in the past 6-7 years that peace prevailed and the population returned to their homes.

Field Code Changed

Participants

Participants were patients with either nodding syndrome or other convulsive epilepsies receiving treatment at any one of the nodding syndrome treatment centres in the seven districts of Lamwo, Kitgum, Pader, Gulu, Amuru, Lira and Oyam. The definition of head nodding and diagnosis of nodding syndrome is in accord with the criteria developed by international consensus during the World Health Organization facilitated meeting on Nodding Syndrome in Kampala, 2012[13]. Head nodding was defined as repetitive, involuntary drops of the head on to the chest in previously normal persons. We included probable and confirmed cases only. Children with other convulsive epilepsies were those with active (at least one in the past year) tonic-clonic or focal jerking epileptic seizures. The diagnosis and classification of epilepsy in this rural community is quite limited and in many cases, categorisation into specific clinical groups is not possible. We therefore only included those with convulsive epilepsies. Participants ~~Those~~ with onset of symptoms outside of the ages 3-18 years were excluded to allow comparability with nodding syndrome patients.

Formatted: Space After: 0 pt, Line spacing: 1.5 lines

The intervention

The nodding syndrome treatment centres in Lamwo, Kitgum and Pader were opened in March 2012 followed by those in Amuru, Gulu, Lira and Oyam in June 2012. Prior to this, clinicians and nurses at each centre underwent a five-day training on the management of nodding syndrome using the specified guideline[14]. The training which also included general principles of epilepsy treatment was provided through didactic lectures, role play, bedside clinical teachings and demonstrations by the same team that developed the guidelines. At the end of the five days, each

For peer review only - http://bmjopen.bmj.com/site/about/guidelines.xhtml

Treatment Outcomes of Nodding Syndrome in Uganda

team returned to their centre and worked with the trainers to initiate provision of care. Other than the centre in Kitgum which is a district hospital (a level V health centre), all the others were health centre III. At each centre, clinical service was led by a medical or psychiatric clinical officer (individuals with a diploma in clinical medicine or psychiatry after three years of training), general and psychiatric nurses, laboratory technicians and either a physiotherapist or occupational therapist. In Kitgum hospital, the team was led by a medical officer (MBChB). These teams were supported by local lay volunteers - village health workers – who coordinated follow up and ambulatory care in homes. In each district, supervisory oversight was provided by a district nodding syndrome focal person, the District Health Officer and the district nodding syndrome committee while nationally, there was a national nodding syndrome coordinator who brought everyone together. Over the next 12 months, each centre received support supervision visits on at least two occasions to maintain skills and attend to issues arising.

Details of the treatment are described elsewhere[10]. In summary, inpatient emergency care was offered to patients with life threatening co-morbidities. Ambulatory and community care was offered to patients without co-morbidities or those with non-life threatening co-morbidities. Sodium valproate was the first-line anticonvulsant starting at 10mg/kg/day in two divided doses and the dose titrated to a maximum of 40mg/kg/day. The patient's family was provided with supplemental food rations every 2-4 weeks. Severely malnourished patients with medical complications were treated as inpatients and those with uncomplicated severe malnutrition were treated as outpatients with ready to use therapeutic feeds. This was provided as Plumpy'Nut® a product of Nutriset, (Normandy, France). Plumpy'Nut is made of peanut paste, vegetable oil, powdered milk and sugar, vitamins (A, B-complex, C, D, E and K) and minerals (calcium, phosphorus, potassium, magnesium, zinc, copper, iron, iodine, sodium and selenium) all combined in a foil pouch. Each 92g pack provides 500 calories. Management of behaviour and emotional difficulties included counselling and referral of those with severe symptoms to mental health services. Other management included physical, speech and language therapy and cognitive stimulation.

Children with other convulsive epilepsies were provided with first-line anticonvulsants (carbamazepine, phenobarbitone, phenytoin or sodium valproate) or continued to receive earlier prescribed anticonvulsants but the dose adjusted appropriately. A new anticonvulsant was introduced if an inappropriate drug was being provided. Anticonvulsants such as oxcarbazepine, lamotrigine, levetiracetam, and topiramate are unavailable in the public health service in Uganda. Families of patients with other convulsive epilepsies were also provided with similar supplemental

For peer review only - http://bmjopen.bmj.com/site/about/guidelines.xhtml

feeding. In addition, parents/carers of both groups of patients were educated on seizures, epilepsy, adherence to antiepileptic drugs and prevention of seizure related injuries.

Sample size

In a preliminary evaluation of the treatment outcomes of nodding syndrome after seven months of intervention, we documented (from parental or carer report) that 5/47 (10%) had achieved seizure freedom (no head nodding or convulsive seizures) for at least 30 days prior to the visit. Using these findings, we estimated that a sample of 432 patients will be sufficient at 5% level of significance and 90% power to detect a 10% increase in this proportion to 20% after 12 months of treatment. Secondly, up to 70% of children with new onset convulsive epilepsies achieve terminal seizure remission with drug treatment.[15, 16] The onset of seizure remission is often evident within the first year of treatment[15]. Using these findings, we estimated that with a sample of 461 nodding syndrome patients and a similar number with other convulsive epilepsies, we will be able to, at 90% power and 5% level of significance, reject the null hypothesis that there is no difference in the proportions of patients with nodding syndrome or other convulsive epilepsies achieving seizure freedom with 12 months therapy. We set to recruit the larger sample.

Study procedures and measurements

~~[16]~~As of 30 June 2013, there were ~~a reported~~ 3,295 patients with probable or confirmed nodding syndrome receiving care at the seven centres. We used proportionate sampling to estimate the number of participants to be recruited from each centre and consecutively recruited patients as they presented until the sample was achieved. Data was collected between 1st July 2013 and 30th September 2013. One of two investigators (RI or BTO) first conducted a day's training on the study procedures followed by a joint clinic with the clinicians at the centre. The local clinical team subsequently worked independently until study completion. Case record forms were completed from data abstracted from pre-intervention records, direct inquiry from parents/carers and on physical exam. The pre-intervention seizure burden, weight and height, and behaviour or emotional difficulty was obtained from records. We defined seizures as head nodding or convulsive seizures and defined seizure burden as the number of clusters of head nodding and/or convulsive seizures per unit time.

In the clinic, parents/carers reported on current seizures, behaviour and emotional difficulties. W~~The w~~eight was measured using a stand on electronic scale while height or length was

For peer review only

measured using a stadiometer. Independence in basic self care (self feeding, dressing and using a toilet), the status of schooling, and ability to appropriately help with culturally and age-appropriate homecare activities (e.g. sweeping the compound) were obtained from the parents or carers. The parents and carers were also asked to provide an overall assessment of improvements or worsening of symptoms over the year on an ordinal scale (markedly improved, some improvement, no improvement or worse).

Outcome measures

The primary outcome was the proportion of patients who had achieved seizure freedom (defined as ≥1 month without seizures [no head nodding and/or convulsive seizures observed by the parent/carer prior to follow up visit]). Secondary outcomes included reduction in seizure burden (reduction in the mean number of clusters of head nods and/or convulsive seizures per unit time), the proportions of patients with independence in basic self care, resolution of behaviour and emotional difficulties, and enrolment in school.

Data management and statistical analysis

Data was collected on case record forms and double-entered into a Microsoft Access 2007 database. Data analysis was performed using STATA version 12.0 (STATA Corp, Tx). The two patient groups were considered as two independent single samples and paired data (before initiation of therapy and at least 12 months later) analysis was performed for each group. Thus, we determined the proportions of patients with nodding syndrome with seizure freedom before and after 12 months and the proportions with the different secondary outcomes. A one sample t-test was used to compare means of normally distributed continuous data, the Mann-Whitney U test for medians of skewed data and McNemar's test for categorical data. ~~The outcomes of patients with nodding syndrome were then compared to those of patients with other convulsive epilepsies.~~ We then examined for ~~variables associated with seizure freedom~~ patient characteristics potentially associated with seizure freedom including duration and age at onset of symptoms, baseline seizure frequency, presence of behaviour and emotional difficulties, whether the child had head nodding only or head nodding plus (other seizures) and antiepileptic drug dose and performed a logistic regression analysis to determine variables independently associated with achieving seizure freedom.

RESULTS

General descriptions

For peer review only - http://bmjopen.bmj.com/site/about/guidelines.xhtml

Treatment Outcomes of Nodding Syndrome in Uganda

A total of 1,322 subjects were screened in six out of the seven districts. Oyam district, which had only eight patients with nodding syndrome, was not visited. Two hundred and fifteen subjects were ineligible. Another 147 were also excluded for different reasons. Thus, 960 participants (484 with nodding syndrome and 476 with other convulsive epilepsies) were available for the study (**figure 1**).

The two groups were of similar age and gender; the mean (SD) age of patients with nodding syndrome was 13.7(3.6) years and that for patients with other convulsive epilepsies was 13.0(2.9) years, *p*=0.998; 281/484 (58.1%) subjects with nodding syndrome and 267/476 (56.1%) with other convulsive epilepsies were male, *p*=0.538. However, participants with nodding syndrome had experienced a longer duration of symptoms (median 5[IQR 3, 6] years) compared to patients with other epilepsies, (median 4 [IQR 2, 6] years), *p*<0.001.

The median daily dose of sodium valproate in patients with nodding syndrome was 16(IQR 12, 21) mg/kg/day with most (298/484, 61.6%) on relatively low doses (<20mg/kg/day). The majority of the patients with other convulsive epilepsies (421/476, 88.5%) were on carbamazepine, phenobarbitone or phenytoin monotherapy. The remaining 55 were either on sodium valproate (40/476, 8.4%) or combinations of the above anticonvulsants (15/476, 3.1%).

Outcomes of interventions

a) Seizures

There was marked reduction in seizures with the intervention; overall, 25% (95% CI 21, 29) of nodding syndrome patients achieved seizure freedom. Both the frequency of head nodding and of convulsive seizures reduced by over 70%. The reduction in seizure burden was even more marked in patients with other convulsive epilepsies; 51% (95% CI 46.4, 55.6) achieved seizure freedom and the overall burden of seizures decreased by 86%, **(table 1)**.

Although the effects of sodium valproate on seizure control in nodding syndrome was evident at relatively low doses, additional patients achieved seizure freedom with dose escalation. Thus, 87/298 (29.2%) patients were seizure free on sodium valproate <20mg/kg/day and an additional 34/186 (18.3%) achieved seizure freedom with dose increases to 20-40mg/kg/day.

For peer review only

For peer review only - http://bmjopen.bmj.com/site/about/guidelines.xhtml

Treatment Outcomes of Nodding Syndrome in Uganda

Table 1 **Pre-interventions features and features at least 12 months after initiation of a symptomatic treatment intervention in patients with nodding syndrome or other convulsive epilepsies**

	Patients with nodding syndrome, N=484			Other convulsive epilepsies, N=476		
	**Pre- intervention status**	**Features ≥ 12 months later**	**P value**	**Pre-intervention status**	**Features ≥ 12 months later**	**P value**
**Patients with seizure freedom*, %**	8 (2%) [95% CI 0.07, 3.2]	121 (25.0%) [95% CI 21.2, 29.1]	<0.001	8 (2%) [95% CI 0.7, 3.3]	243 (51.1%) [95% CI 46.4, 55.6]	<0.001
**Daily clusters of head nods, median (IQR)**	4 (IQR 3, 6)	1 (IQR 0, 2)	<0.001	-	-	-
**Patients with behaviour & emotional difficulties, %**	327/484 (67.6%) [95% CI 63.2, 71.7]	133 (27.5%) [95% CI 23.5, 33.7]	<0.001	250/476 (52.5%) [95% CI 47.9, 57.1]	105 (22.1%) [95% CI 18.4, 26.1]	<0.001
**GMFCS score****						
**1**	185/282 (64.0%)	223/282 (79.1%)	<0.001***	212/288 (73.6%)	239/288 (83.0%)	<0.001***
**2**	58/282 (20.1%)	39/282 (13.8%)		41/288 (14.1%)	39/288 (13.5%)	
**3**	39/288 (13.5%)	39/288 (13.5%)		39/288 (13.5%)	10/288 (3.5%)	
**4 and 5**	39/288 (13.5%)	0 (0)		2/288 (0.7%)	0 (0)	
**Independence in basic self care, %**	174 (36.0%) [95% CI 31.7, 40.4]	402 (83.1%) [95% CI 79.4, 86.3]	< 0.001	206 (43.3%) [95% CI 38.8, 47.9]	397 (83.4%) [95% CI 79.8, 86.6]	<0.001
**Able and performs culturally and age appropriate homecare activities, %**	152 (31.4%) [95% CI 27.2, 37.4]	372 (76.9%) [95% CI 72.8, 80.5]	<0.001	187 (39.3%) [95% CI 34.9, 43.8]	382 (80.3%) [95% CI 76.4, 83.7]	<0.001
**Enrolled at and attending school, %**	107 (22.1%) [95% CI 18.5, 26.1]	193 (39.9%) [95% CI 35.5, 44.4]	<0.001	170 (35.7%) [95% CI 31.4, 40.2]	250 (52.5%) [95% CI 47.9, 57.1]	<0.001

*≥1 month without seizures
**GMFCS=Gross Motor Function Classification Score; N=282; i.e. Only 282 patients with nodding syndrome had paired GMFCS pre and post interventions scores obtained.
***Chi square test for trend with Yate's correction

We repeated diagnostic electroencephalogram (EEG) recordings for three patients with nodding syndrome who were part of the 22 we reported on earlier[6]. The recordings showed clear improvements in background EEG and reductions in previously widespread interictal epileptiform discharges. All three were on sodium valproate 20-25mg/kg/day and were experiencing only occasional convulsive seizures but no head nodding.

Formatted: Indent: Left: 0.5", No bullets or numbering

For peer review only - http://bmjopen.bmj.com/site/about/guidelines.xhtml

Treatment Outcomes of Nodding Syndrome in Uganda

b) Behaviour and emotional difficulties

Behaviour and emotional difficulties were reported in 327(67.6%) participants with nodding syndrome and in 250(52.5%) with other convulsive epilepsies prior to the intervention. Among participants with nodding syndrome, these included aggressive and destructive behaviour (186/484, 39.5%), wandering or running away (113/484, 23.4%) and periods of low mood (114/484, 23.6%). Over the 12 months, the difficulties resolved in 194/327 (59.3%) nodding syndrome patients and in 145/250 (58.0%) patients with other convulsive epilepsies. Improvements were most evident in nodding syndrome patients initially reporting wandering, aggressive and destructive behaviour. Psychotropic drugs (haloperidol) were prescribed for only three patients with severe difficulties and two received anxiolytic drugs. An additional 62(12.8%) nodding syndrome patients, especially those with uncontrolled or worsening seizures, developed new onset behaviour and emotional difficulties; these included 44 (9.1%) aggressive and destructive behaviour, 18(3.7%) wandering, and 21(4.3%) mood problems. Wandering behaviour was uncommon among patients with other convulsive epilepsies in whom impulsive behaviour and hyperactivity were more common.

c) Independence in basic self care

Prior to the intervention, 174/484(36.0%) patients with nodding syndrome were independent in basic self care. This proportion had increased to 402/484(83.1%) by the time of the survey, *p*<0.001. Similar improvements were observed in patients with other convulsive epilepsies. Thus, 397/476(83.4%) of these patients were independent in basic self care at the time of the survey up from 270/476 (56.7%) prior to intervention, p<0.001.

d) School of attendance

A total of 443 patients (193/484, 39.9% with nodding syndrome and 250/476, 52.5% with other convulsive epilepsies) were enrolled in and attending school at the time of the survey. This included 86/484 (17.8%) patients with nodding syndrome and 80/476 (16.8%) patients with other convulsive epilepsies who had returned to school with seizure control and improvements in other symptoms. Although these children had~~ve~~ returned to school, parents reported that 90/193(46.6%) patients with nodding and 76/250(30.4%) patients with other epilepsies were still performing poorly in school.

For peer review only

For peer review only - http://bmjopen.bmj.com/site/about/guidelines.xhtml

Treatment Outcomes of Nodding Syndrome in Uganda

e) Qualitative assessment of improvements by parents and carers

On an ordinal subjective scale, parents felt that 112/484(23.1%) patients with nodding syndrome and 253/476(53.2%) patients with other convulsive epilepsies had improved markedly. Another 325/484(67.2%) patients with nodding syndrome and 194/476(40.8%) with other convulsive epilepsies had some improvement. The number of patients with nodding syndrome who could participate and help their parents with home care tasks increased from 152/484 (31.4%) to 372/484 (76.9%) with the intervention. Only 47/484(9.7%) patients with nodding syndrome and 29/476(6.1%) with other convulsive epilepsies had no improvement in symptoms or became worse over the period of intervention.

Prognostic factors for seizure freedom

While we examined the relationship between gender, age at onset of symptoms, duration of symptoms, baseline seizure frequency, presence of behaviour and emotional difficulties, whether the child had head nodding only or head nodding plus (other seizures), antiepileptic drug dose and achieving seizure freedom, o. Only a lower number of clusters of head nods prior to the intervention (adjusted OR 0.80 [95% CI 0.72-0.88], p<0.001) and response to a lower antiepileptic drug dose (adjusted OR 0.96 (95% CI 0.93, 0.99], p=0.046) were independently associated with achieving seizure freedom.

DISCUSSION

Our study aimed to determine the clinical outcomes and therefore, the effectiveness of a symptomatic treatment intervention for nodding syndrome. We documented substantial clinical and functional improvements with the intervention. The findings suggest that nodding syndrome is probably a reversible encephalopathy. The improvements we observed were however less than that seen in patients with other convulsive epilepsies suggesting that epileptic seizures in nodding syndrome may be less anticonvulsant sensitive compared to seizures in the other convulsive epilepsies.

Although the number proportion of patients with nodding syndrome who achieved seizure freedom was modest, our findings suggest that a treatment package of selected anticonvulsants, psycho-behavioural interventions and nutritional and physical rehabilitation can control seizures, improve function and even reverse some severe functional disability in nodding syndrome. This observation seems to concur with a report from Tanzania in which although seizure freedom was achieved by 2/32 patients treated with phenobarbitone, over 80% had reductions in seizure

For peer review only - http://bmjopen.bmj.com/site/about/guidelines.xhtml

Treatment Outcomes of Nodding Syndrome in Uganda

burden.where symptoms of nodding syndrome completely resolved in four of the original cohort of 62 patients.[9] Even though we did not perform specific cognitive testing or brain imaging to objectively document functional and structural improvements with the intervention, comparisons of a pre-intervention and repeat EEG recordings in three patients with previous recordings demonstrated clear improvements in background EEG and reductions in the previously widespread interictal epileptiform discharges.[6]

Field Code Changed

Clinical trials comparing treatment of seizures in nodding syndrome with sodium valproate to treatment with other anticonvulsants such as lamotrigine or levetiracetam either as monotherapy or as add on therapy may be considered. In addition and especially for patients whose symptoms are either not controlled or became worse on therapy, other strategies may be considered. Epileptic encephalopathy is a possibility especially in patients with severe and persistent symptoms. Can therapy with benzodiazepines, high dose steroids or other immunosuppressant drugs be considered?[17]

The aetiology of nodding syndrome is still unknown. In all three countries where nodding syndrome has been described, it has been associated with infestation by *Onchocerca volvulus*.[1, 7, 18] Uganda is in its second year of twice yearly mass administration of ivermectin (an antimicrofilarial agent active only against the mirofilaria but not the adult parasite). Other strategies that target the both microfiliaria and the adult worms and/or their co-symbiotic bacteria, Wolbachiae, may be considered as potential specific therapy.[19, 20]

Despite these improvements, parents reported that the majority of the 40% children who returned to school continued to perform poorly. There is need to examine whether the continued poor academic performance is due to irreparable brain injury or an underlying ongoing aetio-pathogenic process. To date, there are no systematic studies of cognitive function in nodding syndrome. Such studies will help define areas of functional deficits and document improvements preferably using tools that can be applied across different regions with minimal modification to allow comparison.

We did not apply specific psychiatric diagnostic tools to patients with behaviour and emotional difficulties to be able to make distinct psychiatric diagnoses. A few children with severe difficulties were attended to by the local mental health services and some given psychotropic drugs. The majority of the 194 children in whom behaviour and emotional difficulties resolved however

For peer review only

Treatment Outcomes of Nodding Syndrome in Uganda

improved without psychotropic drugs but with seizure control suggesting that in nodding syndrome, some of these features may be co-morbidities of epilepsy. Wandering behaviour may be an ictal event[6]. Some patients may also have benefitted from the effects of sodium valproate on behaviour; in a recent case series of Ugandan children, Musisi et al documented improvements in some patients receiving antidepressants.[21] Put together, these findings suggest that psychotropic drugs may be considered for some patients with nodding syndrome especially those with severe symptoms.

Study limitations

First, other than head nodding, seizures in nodding syndrome may be similar to seizures in other convulsive epilepsies and over time, head nodding may cease in some patients.[9] This scenario opens room for potential misclassification of disease as the current disease criteria heavily leans on clinical observations. Secondly, we did not perform a prospective study; instead, we relied on patient records for pre-intervention features. Third, we had only limited data on the burden and severity of other co-morbidities such as injuries (e.g. burns) or earlier exposure to acute encephalopathies such as cerebral malaria, meningitis and encephalitis. Fourth, participants had varied periods of exposure to the intervention, a factor that may have affected the estimate of the effect. Fifth, we did not determine compliance to antiepileptic drugs or have reports of adverse effects patients experienced while on treatment. We also did not have a detailed documentation of the nutritional therapy and the cognitive stimulatory activities each child received and did not assess the effect of home environment on outcome. We however limited the effects of such bias by choosing only ~~a~~ few and fairly robust outcome measures.

Failure to conduct a prospective study means that we cannot comment on the incidence of death or on patients who might have discontinued follow up care (e.g. due to deterioration in symptoms, severe motor disability or loss of faith in the treatment) leading to an over estimate of the effect. Such an effect if any is likely minimal. From Ministry of Health epidemiological surveillance reports, only 12 patients with probable nodding syndrome died over the period of observation mostly from seizure related events.

Furthermore, our comparative group – participants with other convulsive epilepsies – were a heterogeneous group with different seizure types and possibly neuropathology, on treatment with different anticonvulsants each with different efficacy, dose and side effects. It would have served us better to recruit a more homogenous group of patients, for example, only patients with

For peer review only - http://bmjopen.bmj.com/site/about/guidelines.xhtml

Treatment Outcomes of Nodding Syndrome in Uganda

generalised seizures on treatment with a single anticonvulsant. However, in this rural community, the diagnosis of epilepsy is only limited to clinical features obtained on history and clinical observations by clinicians with limited training. Despite this weakness, our results clearly demonstrate that the outcome of nodding syndrome is different from that of the combined heterogeneous group of patients with the other convulsive epilepsies.

Conclusions

The symptoms and psychomotor functioning of patients with nodding syndrome improve with symptomatic treatments suggesting that nodding syndrome is probably a reversible epileptic encephalopathy. Symptom reversibility may depend on the timing of interventions. Uncontrolled epileptic seizures may be a major contributor to the neuro-cognitive decline and disability in this syndrome. Further studies to elucidate these findings recommended.

For peer review only

For peer review only - http://bmjopen.bmj.com/site/about/guidelines.xhtml

Treatment Outcomes of Nodding Syndrome in Uganda

Author's contributions

RI, HN, CA, BBM, AKM, ROO, AKM, ADM and BTO designed the intervention and the study and participated in supervising patient care. RI and HN performed the data analysis. RI wrote the first draft and all participated in data interpretation and provided a critical review of the manuscript.

Funding

The study was funded by the Government of Uganda. Dr Idro is partly supported by the Wellcome Trust through a Director's discretionary research funds. The funding agency had no role in data collection, analysis and interpretation of results and the decision to submit for publication.

Competing interests

There are no conflicts of interest to report.

Ethics approval

Makerere University School of Medicine Research and Ethics Committee

Data sharing statement

No additional data are available.

Acknowledgements

We would like the thank Local District Health Services in Lamwo, Kitgum, Pader, Amuru, Gulu, and Lira who made the study possible, Keron Ssebyala who managed the data and Prof Gretchen Birbeck, who critically reviewed the first draft of the this manuscript and offered helpful comments to improve it.

For peer review only

For peer review only - http://bmjopen.bmj.com/site/about/guidelines.xhtml

Treatment Outcomes of Nodding Syndrome in Uganda

Reference

1. Tumwine JK, Vandemaele K, Chungong S, Richer M, Anker M, Ayana Y, et al. Clinical and epidemiologic characteristics of nodding syndrome in Mundri County, southern Sudan. African health sciences. 2012; 12(3): 242-8.
2. Spencer PS, Vandemaele K, Richer M, Palmer VS, Chungong S, Anker M, et al. Nodding syndrome in Mundri county, South Sudan: environmental, nutritional and infectious factors. African health sciences. 2013; 13(2): 183-204.
3. Nyunguraa JL, Akimb, T., Lakoc, A., Gordond, A., Lejenge, L., William, G. Investigation into the Nodding syndrome in Witto Payam, Western Equatoria State, 2010. Southern Sudan Medical Journal. 2011 4(1): 3-6.
4. Sejvar JJ, Kakooza AM, Foltz JL, Makumbi I, Atai-Omoruto AD, Malimbo M, et al. Clinical, neurological, and electrophysiological features of nodding syndrome in Kitgum, Uganda: an observational case series. Lancet neurology. 2013; 12(2): 166-74.
5. Foltz JL, Makumbi I, Sejvar JJ, Malimbo M, Ndyomugyenyi R, Atai-Omoruto AD, et al. An Epidemiologic Investigation of Potential Risk Factors for Nodding Syndrome in Kitgum District, Uganda. PloS one. 2013; 8(6): e66419.
6. Idro R, Opoka RO, Aanyu HT, Kakooza-Mwesige A, Piloya-Were T, Namusoke H, et al. Nodding syndrome in Ugandan children--clinical features, brain imaging and complications: a case series. BMJ open. 2013; 3(5).
7. Winkler AS, Friedrich K, Konig R, Meindl M, Helbok R, Unterberger I, et al. The head nodding syndrome--clinical classification and possible causes. Epilepsia. 2008; 49(12): 2008-15.
8. Winkler AS, Friedrich K, Meindl M, Kidunda A, Nassri A, Jilek-Aall L, et al. Clinical characteristics of people with head nodding in southern Tanzania. Tropical doctor. 2010; 40(3): 173-5.
9. Winkler AS, Wallner B, Friedrich K, Pfausler B, Unterberger I, Matuja W, et al. A longitudinal study on nodding syndrome--a new African epilepsy disorder. Epilepsia. 2014; 55(1): 86-93.
10. Idro R, Musubire KA, Byamah Mutamba B, Namusoke H, Muron J, Abbo C, et al. Proposed guidelines for the management of nodding syndrome. Afr Health Sci. 2013; 13(2): 219-32.
11. Iyengar PJ, Wamala J, Ratto J, Blanton C, Malimbo M, Lukwago L, et al. Prevalence of nodding syndrome--Uganda, 2012-2013. MMWR Morbidity and mortality weekly report. 2014; 63(28): 603-6.
12. Wendo C. Northern Uganda humanitarian crisis shocks UN chief. Rebels in northern districts have left people trapped in hunger, disease, poverty, and fear. Lancet. 2003; 362(9398): 1818.
13. WHO. International Scientific Meeting on Nodding Syndrome. Geneva: World Health Organization; 2012.
14. Idro R, Musubire KA, Byamah Mutamba B, Namusoke H, Muron J, Abbo C, et al. Proposed guidelines for the management of nodding syndrome. African health sciences. 2013; 13(2): 219-32.
15. Hauser E, Freilinger M, Seidl R, Groh C. Prognosis of childhood epilepsy in newly referred patients. Journal of child neurology. 1996; 11(3): 201-4.
16. Berg AT, Shinnar S, Levy SR, Testa FM, Smith-Rapaport S, Beckerman B, et al. Two-year remission and subsequent relapse in children with newly diagnosed epilepsy. Epilepsia. 2001; 42(12): 1553-62.
17. Parisi P, Spalice A, Nicita F, Papetti L, Ursitti F, Verrotti A, et al. "Epileptic encephalopathy" of infancy and childhood: electro-clinical pictures and recent understandings. Current neuropharmacology. 2010; 8(4): 409-21.
18. Nodding syndrome - South Sudan, 2011. MMWR Morb Mortal Wkly Rep. 2012; 61(3): 52-4.
19. Hoerauf A, Specht S, Buttner M, Pfarr K, Mand S, Fimmers R, et al. Wolbachia endobacteria depletion by doxycycline as antifilarial therapy has macrofilaricidal activity in onchocerciasis: a randomized placebo-controlled study. Medical microbiology and immunology. 2008; 197(3): 295-311.

For peer review only

20. Hoerauf A, Mand S, Adjei O, Fleischer B, Buttner DW. Depletion of wolbachia endobacteria in Onchocerca volvulus by doxycycline and microfilaridermia after ivermectin treatment. Lancet. 2001; 357(9266): 1415-6.
21. Musisi S, Akena D, Nakimuli-Mpungu E, Abbo C, Okello J. Neuropsychiatric perspectives on nodding syndrome in northern Uganda: a case series study and a review of the literature. African health sciences. 2013; 13(2): 205-18.

For peer review only

For peer review only - http://bmjopen.bmj.com/site/about/guidelines.xhtml

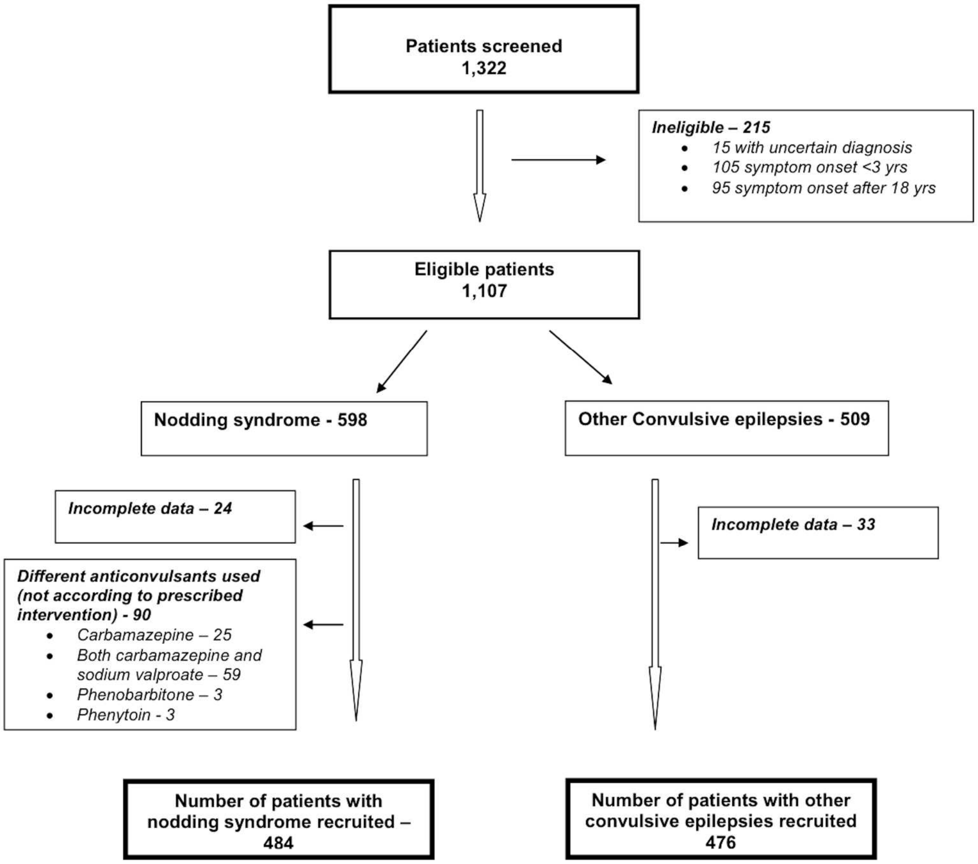

206x201mm (300 x 300 DPI)

STROBE 2007 (v4) Statement—Checklist of items that should be included in reports of *cross-sectional studies*

Section/Topic	Item #	Recommendation	Reported on page #
**Title and abstract**	1	(*a*) Indicate the study's design with a commonly used term in the title or the abstract	1
		(*b*) Provide in the abstract an informative and balanced summary of what was done and what was found	2
**Introduction**			
Background/rationale	2	Explain the scientific background and rationale for the investigation being reported	4
Objectives	3	State specific objectives, including any prespecified hypotheses	4
**Methods**			
Study design	4	Present key elements of study design early in the paper	5
Setting	5	Describe the setting, locations, and relevant dates, including periods of recruitment, exposure, follow-up, and data collection	5-7
Participants	6	(*a*) Give the eligibility criteria, and the sources and methods of selection of participants	5-6
Variables	7	Clearly define all outcomes, exposures, predictors, potential confounders, and effect modifiers. Give diagnostic criteria, if applicable	5, 7, 8
Data sources/ measurement	8*	For each variable of interest, give sources of data and details of methods of assessment (measurement). Describe comparability of assessment methods if there is more than one group	6-7
Bias	9	Describe any efforts to address potential sources of bias	*6*
Study size	10	Explain how the study size was arrived at	7
Quantitative variables	11	Explain how quantitative variables were handled in the analyses. If applicable, describe which groupings were chosen and why	8
Statistical methods	12	(*a*) Describe all statistical methods, including those used to control for confounding	8
		(*b*) Describe any methods used to examine subgroups and interactions	N/A
		(*c*) Explain how missing data were addressed	Figure 1
		(*d*) If applicable, describe analytical methods taking account of sampling strategy	8
		(*e*) Describe any sensitivity analyses	N/A
**Results**			

For peer review only

Participants	13*	(a) Report numbers of individuals at each stage of study—eg numbers potentially eligible, examined for eligibility, confirmed eligible, included in the study, completing follow-up, and analysed	9, Figure 1
		(b) Give reasons for non-participation at each stage	9, Figure 1
		(c) Consider use of a flow diagram	Figure 1
Descriptive data	14*	(a) Give characteristics of study participants (eg demographic, clinical, social) and information on exposures and potential confounders	9
		(b) Indicate number of participants with missing data for each variable of interest	Figure 1, table 1
Outcome data	15*	Report numbers of outcome events or summary measures	9-12, table 1
Main results	16	(*a*) Give unadjusted estimates and, if applicable, confounder-adjusted estimates and their precision (eg, 95% confidence interval). Make clear which confounders were adjusted for and why they were included	11, Table 1,
		(*b*) Report category boundaries when continuous variables were categorized	9-12, table 1
		(*c*) If relevant, consider translating estimates of relative risk into absolute risk for a meaningful time period	N/A
Other analyses	17	Report other analyses done—eg analyses of subgroups and interactions, and sensitivity analyses	N/A
**Discussion**			
Key results	18	Summarise key results with reference to study objectives	12
Limitations	19	Discuss limitations of the study, taking into account sources of potential bias or imprecision. Discuss both direction and magnitude of any potential bias	14
Interpretation	20	Give a cautious overall interpretation of results considering objectives, limitations, multiplicity of analyses, results from similar studies, and other relevant evidence	12-13
Generalisability	21	Discuss the generalisability (external validity) of the study results	12, 14
**Other information**			
Funding	22	Give the source of funding and the role of the funders for the present study and, if applicable, for the original study on which the present article is based	15

*Give information separately for cases and controls in case-control studies and, if applicable, for exposed and unexposed groups in cohort and cross-sectional studies.

Note: An Explanation and Elaboration article discusses each checklist item and gives methodological background and published examples of transparent reporting. The STROBE checklist is best used in conjunction with this article (freely available on the Web sites of PLoS Medicine at http://www.plosmedicine.org/, Annals of Internal Medicine at http://www.annals.org/, and Epidemiology at http://www.epidem.com/). Information on the STROBE Initiative is available at www.strobe-statement.org.

For peer review only
